# Orthogonal Coordination Chemistry of PTA toward Ru(II) and Zn(II) (PTA = 1,3,5-Triaza-7-phosphaadamantane) for the Construction of 1D and 2D Metal-Mediated Porphyrin Networks

**DOI:** 10.1021/acs.inorgchem.0c00080

**Published:** 2020-02-26

**Authors:** Federica Battistin, Alessio Vidal, Paolo Cavigli, Gabriele Balducci, Elisabetta Iengo, Enzo Alessio

**Affiliations:** Department of Chemical and Pharmaceutical Sciences, University of Trieste, Via L. Giorgieri 1, 34127 Trieste, Italy

## Abstract

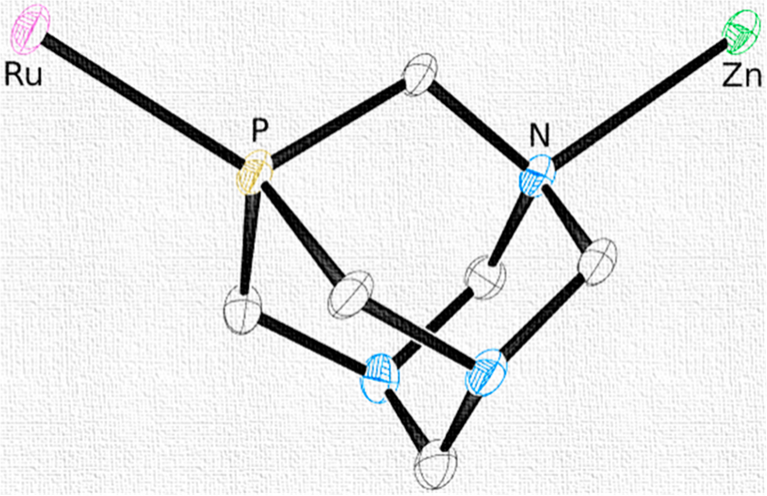

This work demonstrates that PTA (1,3,5-triaza-7-phosphaadamantane) behaves as an orthogonal ligand between Ru(II) and Zn(II), since it selectively binds through the P atom to ruthenium and through one or more of the N atoms to zinc. This property of PTA was exploited for preparing the two monomeric porphyrin adducts with axially bound PTA, [Ru(TPP)(PTA-κ*P*)_2_] (**1**, TPP = *meso*-tetraphenylporphyrin) and [Zn(TPP)(PTA-κ*N*)] (**3**). Next, we prepared a number of heterobimetallic Ru/Zn porphyrin polymeric networks—and two discrete molecular systems—mediated by *P,N*-bridging PTA in which either both metals reside inside a porphyrin core, or one metal belongs to a porphyrin, either Ru(TPP) or Zn(TPP), and the other to a complex or salt of the complementary metal (i.e., *cis,cis,trans*-[RuCl_2_(CO)_2_(PTA-κ*P*)_2_] (**5**), *trans*-[RuCl_2_(PTA-κ*P*)_4_] (**7**), Zn(CH_3_COO)_2_, and ZnCl_2_). The molecular compounds **1**, **3**, *trans*-[{RuCl_2_(PTA-κ^2^*P,N*)_4_}{Zn(TPP)}_4_] (**8**), and [{Ru(TPP)(PTA-κ*P*)(PTA-κ^2^*P,N*)}{ZnCl_2_(OH_2_)}] (**11**), as well as the polymeric species [{Ru(TPP)(PTA-κ^2^*P,N*)_2_}{Zn(TPP)}]_∞_ (**4**), *cis,cis,trans*-[{RuCl_2_(CO)_2_(PTA-κ^2^*P,N*)_2_}{Zn(TPP)}]_∞_ (**6**), *trans*-[{RuCl_2_(PTA-κ^2^*P,N*)_4_}{Zn(TPP)}_2_]_∞_ (**9**), and [{Ru(TPP)(PTA-κ^3^*P,2N*)_2_}{Zn_9_(CH_3_COO)_16_(CH_3_OH)_2_(OH)_2_}]_∞_ (**10**), were structurally characterized by single crystal X-ray diffraction. Compounds **4**, **6**, **9**, and **10** are the first examples of solid-state porphyrin networks mediated by PTA. In **4**, **6**, **8**, **9**, and **11** the bridging PTA has the κ^2^*P,N* binding mode, whereas in the 2D polymeric layers of **10** it has the triple-bridging mode κ^3^*P*,2*N*. The large number of compounds with the six-coordinate Zn(TPP) (the three polymeric networks of **4**, **6** and **9**, out of five compounds) strongly suggests that the stereoelectronic features of PTA are particularly well-suited for this relatively rare type of coordination. Interestingly, the similar 1D polymeric chains **4** and **6** have different shapes (zigzag in **4** vs “Greek frame” in **6**) because the {*trans*-Ru(PTA-κ^2^*P,N*)_2_} fragment bridges two Zn(TPP) units with *anti* geometry in **4** and with *syn* geometry in **6**. Orthogonal “Greek frame” 1D chains make the polymeric network of **9**. Having firmly established the binding preferences of PTA toward Ru(II) and Zn(II), we are confident that in the future a variety of Ru/Zn solid-state networks can be produced by changing the nature of the partners. In particular, there are several inert Ru(II) compounds that feature two or more P-bonded PTA ligands that might be exploited as connectors of well-defined geometry for the rational design of solid-state networks with Zn–porphyrins (or other Zn compounds).

## Abstract 2

This work demonstrates, through the X-ray structural characterization of several polymeric Ru/Zn networks, that PTA (1,3,5-triaza-7-phosphaadamantane) behaves as an orthogonal ligand between Ru(II) and Zn(II). In fact, PTA selectively binds through the P atom to ruthenium and through one or more of the N atoms to zinc.

## Introduction

**Chart 1:**
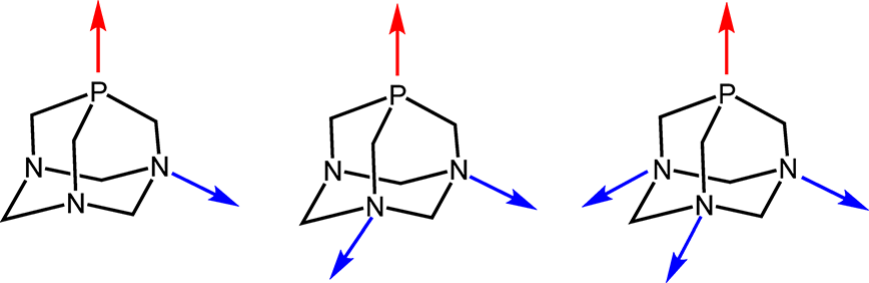
Schematic Structure of PTA and Its Possible Coordination Modes as a Bridging Ligand through P (Red Arrow) and through N Atoms (Blue Arrow)a

The cage-like 1,3,5-triaza-7-phosphaadamantane (PTA, Chart 1), is an amphiphilic, air-stable, neutral ligand of low steric demand (cone angle 103°) characterized by a high solubility in water (ca. 235 g/L). For this reason, PTA and related species—whose coordination chemistry has been thoroughly reviewed by Peruzzini and co-workers—have been largely investigated as supporting ligands for applications in homogeneous aqueous biphasic catalysis and medicinal inorganic chemistry.^1−3^

a Left: (κ^2^*P,N*). Center: (κ^3^*P,2N*). Right: (κ^4^*P,3N*).

PTA typically binds strongly to most transition metal ions through the soft P atom in a monodentate fashion (PTA-κ*P*). However, having also three hard N donor atoms, it is actually a heteropolytopic PN_3_ ligand and might also potentially bridge two or more metal ions with different HSAB preferences (Chart 1).^1^

The bridging κ*P,N* coordination mode of PTA was found to be rather common, even though its first example, the heterobimetallic coordination polymer [{RuCp(dmso-κ*S*)(PTA-κ^2^*P,N*)_2_}{AgCl_2_}]_∞_, was described by Romerosa, Peruzzini and co-workers only in 2005.^4,5^ Subsequently, Romerosa and co-workers described a series of water-soluble 1D ruthenium–metal coordination polymers featuring a Ru–CN–Ru–M backbone (M = Au, Ni, Cd, Co).^6^ They are formed by inert [{RuCp(PTA)_2_}(μ-CN){RuCp(PTA)_2_}]^+^ moieties connected at both ends through bridging PTA-κ^2^*P,N* ligands to metal anions (i.e., [Au(CN)_4_]^−^, [NiCl_3_]^−^, [CdCl_3_]^−^, [CoCl_3_]^−^). In addition, besides two discrete molecular entities featuring a {Re(III)(PTA-κ^2^*P,N*)M(II)} moiety (M = Cu, Zn),^7^ many other examples of PTA-driven polymeric networks with Ag(I),^8,9^ and Cu(I)^10^—often with different ancillary ligands—were reported, mainly by Kirillov, Pombeiro and co-workers. In these structures PTA assumes double (κ^2^*P,N*), triple (κ^3^*P,2N*), and even quadruple (κ^4^*P,3N*) bridging coordination modes. An example of homometallic mixed-valence Cu(I/II) polymeric network, in which PTA binds to Cu(I) with the soft P atom and to Cu(II) with the hard N atom was also described.^11^ Taken together, the results with Ag(I) and Cu(I) suggest that these metal ions are rather promiscuous toward PTA, without a marked preference for N- or P-bonding.

There are instead relatively few examples of complexes containing exclusively N-bonded PTA (PTA-κ*N*). They concern hard metal ions such as Mn(II) and Co(II),^12,13^ or the d^10^ metal ion Zn(II). After the first Zn–PTA complex—the distorted tetrahedral [ZnCl_2_(PTA-κ*N*)_2_]—was described in 2009 by Pombeiro and co-workers,^14^ Reek, Kleij et al. investigated the reactivity of PTA with a number of square-planar Zn(salphen) complexes (salphen = *N*,*N*′-bis(salicylidene)imine-1,2-phenylenediamine) in the context of supramolecular catalysis. It was found that PTA binds to zinc exclusively through the N atoms and can act as a bridge between two or even three Zn(salphen) units, giving [{Zn(salphen)}_2_(PTA-κ^2^*N*)] and [{Zn(salphen)}_3_(PTA-κ^3^*N*)] adducts in which the zinc ions have a distorted square planar geometry.^15^

In the past we and others have explored the coordination chemistry of PTA toward Ru compounds (where it binds through P exclusively).^16,17^ We have also largely exploited the axial coordination of Ru and Zn porphyrins toward polydentate pyridyl ligands for the construction of numerous supramolecular assemblies.^18,19^ Considering that, according to the literature, PTA binds always through P to ruthenium and through N to zinc, we reasoned that it might be exploited as an orthogonal bridging ligand for the preparation of heterobimetallic supramolecular assemblies and/or polymeric networks containing Ru– and Zn–porphyrins. In addition, the presence of PTA might improve their solubility in water or at least in protic solvents.

Phosphine ligands have high association constants with Ru–porphyrins, in the range of 10^6^ to 10^8^ M^–1^,^20^ whereas N ligands, in particular hard tertiary amines, have lower constants. For example, it has been reported that Ph_2_P(CH_2_)_2_NEt_2_ binds to ruthenium porphyrins exclusively through P and the NEt_2_ group remains dangling.^21^ Zn–porphyrins make less robust axial bonds with N-ligands (compared to Ru), that depend also on N hybridization. For example, the association constant of pyridine with Zn(TPP) (TPP = *meso*-tetraphenylporphyrin) was found to be 7.7 × 10^3^ M^–1^ (CH_2_Cl_2_, 25 °C), whereas under the same—or very similar—conditions amines (including tertiary amines) have ca. 10-fold larger association constants.^22,23^ By comparison, hexamethylenetetramine (HTMA)—the all-nitrogen analogue of PTA—was found to make stronger axial bonds with Zn–porphyrins compared to pyridyl functions (probably also because of its low steric demand),^24^ and binding constants in the range 10^5^ to 10^6^ M^–1^ were measured for the axial N-binding of PTA to square planar Zn(salphen) complexes in toluene.^15^

The interactions of PTA with Ru– and Zn–porphyrins have not been investigated before. Thus, in this work we first established the coordination mode of this ligand toward the neutral model metallo-porphyrins [Ru(TPP)(CO)] and Zn(TPP), obtaining the monomeric adducts [Ru(TPP)(PTA-κ*P*)_2_] (**1**) and [Zn(TPP)(PTA-κ*N*)] (**3**), in which PTA is axially bound to the metal inside the porphyrin. Then, we prepared and structurally characterized a number of heterobimetallic Ru/Zn porphyrin polymeric networks mediated by *P,N*-bridging PTA (PTA-κ^2^*P,N*) and, in one case, (PTA-κ^3^*P*,*2N*). In such assemblies either both metal centers reside inside a porphyrin core or one of the two belongs to a coordination compound. Our findings demonstrate that indeed PTA behaves as a selective orthogonal ligand, binding to Ru exclusively through the P atom and to Zn exclusively through the N atoms.

## Results and Discussion

### Reactivity of [Ru(TPP)(CO)] toward PTA

It is well-known from the literature that whereas pyridine or azole ligands (N) replace the labile solvent molecule *trans* to CO in [Ru(por)(CO)(S)] compounds (e.g., por = TPP; S = MeOH or EtOH, typically not indicated in the formula), affording derivatives of the general formula [Ru(por)(CO)(N)],^25−28^ most phosphine and phosphite ligands (P) under mild conditions replace easily also the carbonyl ligand yielding disubstituted compounds of formula [Ru(por)(P)_2_].^20,29−35^ Monosubstituted [Ru(por)(CO)(P)] intermediates with tertiary phosphines have been occasionally isolated,^31^ whereas in most other cases, due to the weakening of the carbonyl ligand by the phosphine *trans* effect, they could not be isolated but were characterized spectroscopically in solution.^20a^ To our knowledge, with the exception of a private communication from Sanders and co-workers,^36^ no X-ray structure of such an intermediate has been reported yet.

**Figure 1:**
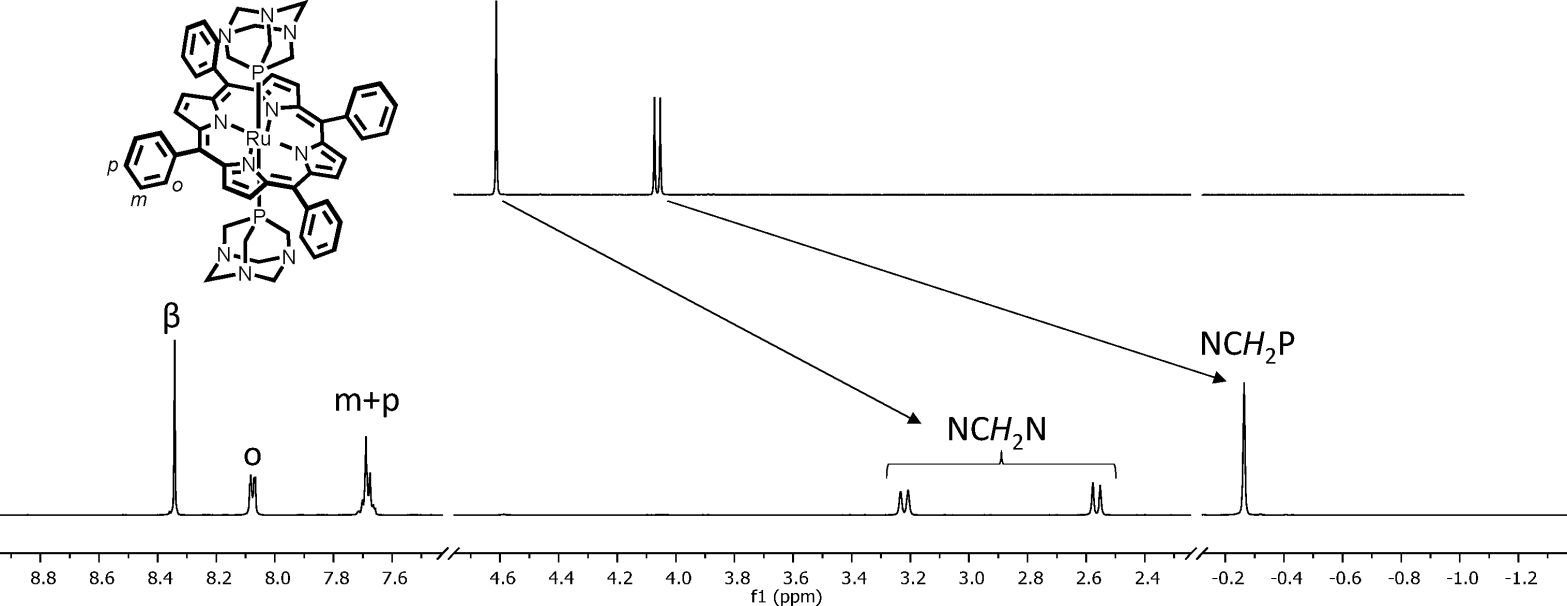
^1^H NMR spectra (CDCl_3_) of PTA (top) and [Ru(TPP)(PTA-κ*P*)_2_] (**1**) (bottom).

We found that PTA reacts with [Ru(TPP)(CO)] as do most other phosphines. When treated with a slight excess of PTA in chloroform solution at room temperature, [Ru(TPP)(CO)] rapidly affords [Ru(TPP)(PTA-κ*P*)_2_] (**1**) in high yield. Axial coordination of two *trans* PTA moieties, bound through the P atom, was clearly evident from NMR spectroscopy (Figure 1). The ^31^P resonance, which is not significantly influenced by the porphyrin shielding cone, occurs as a singlet at −50.6 ppm, i.e., in the typical region for mutually *trans* PTAs coordinated to Ru(II).^16c^ The ^1^H resonances of the PTA methylene protons, and those of the PCH_2_N protons in particular, are shifted to lower frequencies compared to free PTA because the protons fall in the shielding cone of the porphyrin. Thus, the NCH_2_N protons resonate as two well-resolved doublets (6H each) at 3.21 e 2.55 ppm,^37^ whereas the PCH_2_N protons, closer to the macrocycle, give a singlet (12H) at −0.26 ppm. The assignments were confirmed by the HSQC spectrum (Figure S3), since the corresponding carbon atoms have characteristic and well-resolved resonances that are only marginally affected by coordination.^1^ The βH singlet of TPP is shifted to lower frequencies by ca. 0.35 ppm by the replacement of CO with two PTAs. Even though the chemical shifts of the phenyl signals are not particularly affected, by virtue of the increased symmetry the *o*H resonance—that was split into two well-resolved doublets in the spectrum of [Ru(TPP)(CO)]—is a sharp doublet in that of [Ru(TPP)(PTA-κ*P*)_2_] (Figure 1 and Figure S1). Consistent with what found with similar [Ru(por)(P)_2_] adducts,^20,29−35^ the Soret band in the electronic absorption spectrum of **1** is considerably red-shifted compared to [Ru(TPP)(CO)] (431 vs 408 nm).

**Figure 2:**
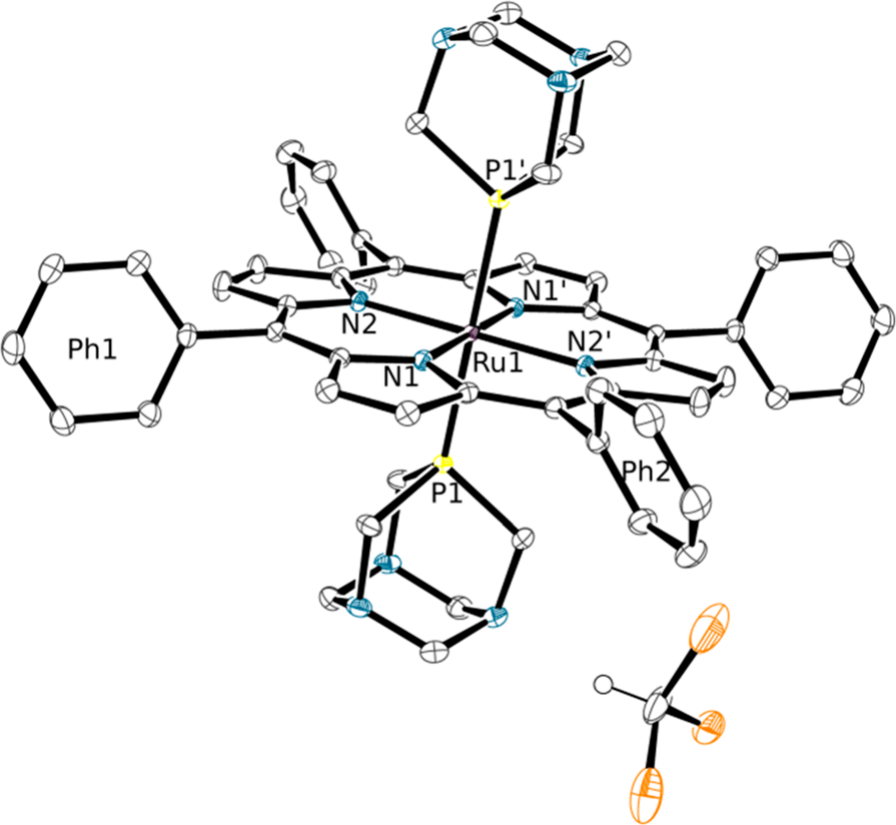
ORTEP representation (50% probability ellipsoids) of the solid state molecular structure of [Ru(TPP)(PTA-κ*P*)_2_]·2CHCl_3_ (**1**·2CHCl_3_). Primed atoms are symmetry mates via (1 – *x*, 1 – *y*, 1 – *z*). Only one of the two symmetry related CHCl_3_ crystallization molecules is shown. The H atoms of the complex are omitted for clarity. The Ph1 and Ph2 labels allow one to visualize on the figure the dihedral angles reported in Table S2.

The geometry of **1** was confirmed by single crystal X-ray analysis (Figure 2). The Ru–P distance in **1** compares well with those in similar [Ru(por)(P)_2_] compounds as well as with those in Ru(II) coordination compounds that feature the {*trans*-Ru(PTA-κ*P*)_2_} fragment (Table 1).^16,20,31^

**Table 1:** Ru–(PTAκ*P*) Bond Lengths in the X-ray Structurally Characterized Compounds

compound	Ru–P distance (Å)	ref
[Ru(TPP)(PTA-κ*P*)_2_] (**1**)	2.3253(7)	this work
[Ru(TPP)(dpm)_2_] (dpm = diphenylphosphinomethane)	2.398(3)	(31a)
[Ru(OEP)(PPh_3_)_2_] (OEP = octaethylporphyrin)	2.428 (average)	(31c)
[Ru(OEP)(dpap)_2_] (dpap = diphenyl phenylacetylene phosphine)	2.3777(5)	(20c)
[Ru(DPP)(dpap)_2_] (DPP = 5,15-bis(3′,5′-di-*tert*-butyl)phenyl-2,8,12,18-tetraethyl-3,7,13,17-tetramethylporphyrin)	2.340(5)–2.3623(10)	(20a)
[Ru(TPP)(dpap)_2_]	2.3597(10)–2.3784(10)	(20c)
Ru(II) compounds with {*trans*-Ru(PTA-κ*P*)_2_} fragment	2.290–2.400	(16c)

Finally, compound **1** was rapidly obtained at room temperature also upon addition of two equiv of PTA to a CDCl_3_ solution of [Ru(TPP)(CO)(py)] (py = pyridine), thus demonstrating that, besides ethanol, PTA also readily replaces axially bound pyridine.

Regretfully, compound **1** was found to be completely insoluble in water, even at acidic pH where protonation of PTA would be expected to improve solubility. For instance, the Ru(0) cluster Ru_3_(CO)_9_(PTA)_3_ can be extracted from a chloroform solution into acidic water (pH < 4).^38^

An NMR titration of PTA into a CDCl_3_ solution of [Ru(TPP)(CO)] allowed us to detect the resonances of the elusive intermediate species [Ru(TPP)(CO)(PTA-κ*P*)] (**2**). The PTA singlet of **2** in the ^31^P{^1^H}NMR spectrum (Figure S4) is remarkably shifted compared to **1** and falls at −60.5 ppm, i.e. in the typical spectral region of PTA *trans* to CO in Ru(II) compounds.^16^ The ^1^H NMR features of **2** (Figure S5) are rather similar to those of **1** in terms of chemical shifts, the most noticeable difference being the split resonance of the *o*H and *m*H protons due to the absence of the macrocycle mirror plane (as in the precursor) that makes the α- and β-side of the porphyrin inequivalent. The solution CO stretching frequency in **2** falls at 1989 cm^–1^.^39^

### Interaction of PTA with Zn(TPP)

The interaction of PTA with the model zinc porphyrin Zn(TPP) was investigated in chloroform solution. The occurrence of the axial binding of PTA to Zn(TPP) was evident from an NMR titration, in which the PTA/Zn(TPP) ratio ranged from 0.5 to 5. For each PTA/Zn ratio the ^31^P{^1^H} resonance of PTA occurred as a singlet at ca. −102.1 ppm (i.e., the same chemical shift of free PTA). Whereas the ^1^H resonances of Zn(TPP) were only slightly affected, those of PTA were broadened and shifted to lower frequencies compared to the free ligand (Figure S6). At PTA/Zn(TPP) = 0.5, the PTA protons gave two equally intense broad resonances, a singlet at ca. 0.9 ppm and a doublet centered at ca. 0.1 ppm. Upon increasing the PTA/Zn ratio, the upfield shift of the PTA resonances progressively decreased.

The NMR findings are consistent with the occurrence of relatively weak and reversible axial interactions between PTA and Zn(TPP), in an equilibrium that is fast on the NMR time scale: the chemical shifts of the PTA resonances are a weighed average between those of PTA axially bound to Zn(TPP), and thus upfield shifted, and those of the free ligand. Actually, assuming that such interaction involves the N atoms of PTA, multiple equilibria can occur as in the case of Zn(salphen)–(PTA-κ*N*) adducts, where PTA can bind axially up to three Zn(salphen) units.^15^ In addition, even though zinc porphyrins are expected to bind preferentially one axial N ligand making square pyramidal adducts, the formation of octahedral products with two axial ligands is not uncommon and cannot be excluded.^24,40−45^ Thus, the NMR spectrum is expected to depend also on the concentration and temperature, in addition to the PTA/Zn ratio. To be noted that, consistent with N-coordination of PTA to Zn(TPP) (and contrary to what observed for **1**), in the ^1^H NMR spectrum at high Zn(TPP)/PTA ratio the resonance of the NCH_2_N protons is shifted more upfield than the NCH_2_P resonance (Figures S6 and S7).

**Figure 3:**
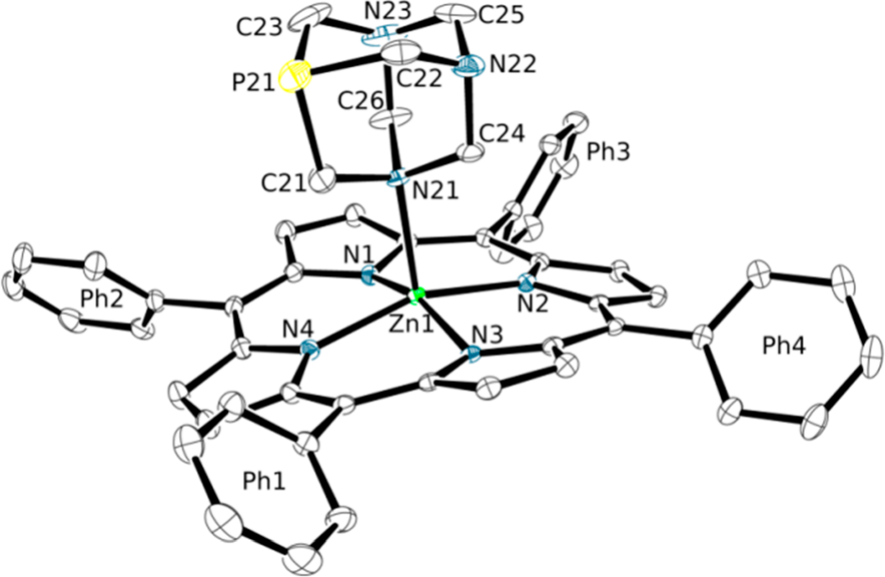
ORTEP representation (50% probability ellipsoids) of the crystal structure of complex [Zn(TPP)(PTA-κ*N*)]·H_2_O·CHCl_3_ (**3**·H_2_O·CHCl_3_). For the sake of clarity, H atoms, one disordered water, and one CHCl_3_ crystallization molecule are omitted. The Ph1–Ph4 labels allow one to visualize on the figure the dihedral angles reported in Table S3.

Slow diffusion of diethyl ether onto the chloroform solution of the PTA/Zn(TPP) = 0.5 mixture afforded crystals of the discrete [Zn(TPP)(PTA-κ*N*)] adduct (**3**), whose X-ray structure is shown in Figure 3. As clear also from the lattice representation (Figure S13), zinc is five-coordinate (i.e., binds to a single PTA molecule), and each PTA is bound to a single Zn(TPP) unit.

As already observed for N-coordination of PTA, as well as for protonation and alkylation, the N21–C bond distances are slightly elongated—compared to the other N–C distances—upon coordination to Zn. The axial Zn–N(PTA) bond length is longer than in the distorted tetrahedral complex [ZnCl_2_(PTA-κ*N*)_2_]^14^ (and in similar complexes with O=PTA and S=PTA),^46^ but compares rather well with those found in the square-pyramidal Zn(salphen)–(PTA-κ*N*) adducts where PTA occupies the axial position (Table 2).^15^

**Table 2:** Zn–(PTA-κ*N*) and Zn–(HTMA-κ*N*) Bond Lengths in the X-ray Structurally Characterized Compounds

compound	axial Zn–N distance (Å)	ref
[Zn(TPP)(PTA-κ*N*)] (**3**)	2.186(2)	this work
[ZnCl_2_(PTA-κ*N*)_2_]	2.055(3)–2.101(3)	(14)
[ZnCl_2_(O = PTA-κ*N*)(OH_2_)]	2.0931(10)	(46)
[{Zn(salphen)}_2_(PTA-κ^2^*N*)]	2.103(6), 2.172(7)	(15)
[{Zn(salphen)}_3_(PTA-κ^3^*N*)]	2.194(4), 2.201(4), 2.200(3)	(15)
[Zn(TOHPP)(HTMA)_2_] (TOHPP = tetra(4-hydroxyphenyl)porphyrin)	2.520(2)	(24)
[Zn(TCPP)(HTMA)_2_] (TCPP = tetra(4-carboxyphenyl)porphyrin)	2.510(2)	(24)
[Zn(TPyP)(HTMA)] (TPyP = tetra(4′-pyridyl)porphyrin)	2.189(3)	(24)

### PTA-Bridged Heterobimetallic Ru/Zn Compounds

The above results indicate that the axial binding of PTA toward Ru– and Zn–porphyrins is truly orthogonal and might be exploited to create heterodinuclear supramolecular porphyrin assemblies connected by bridging PTA moieties.

Thus, we investigated the interaction between [Ru(TPP)(PTA-κ*P*)_2_] (**1**) and Zn(TPP). An NMR titration of Zn(TPP) into a CDCl_3_ solution of **1**, in which the Zn(TPP)/**1** ratio ranged from 1 to 4 (Figure S8) showed that the PTA resonances were broadened and gradually shifted to lower frequencies upon increasing the number of Zn(TPP) equivalents. Conversely, the resonances of the two porphyrins, as well as the ^31^P{^1^H} resonance of PTA, were only marginally affected. These findings are consistent with the establishment of an axial Zn–(PTA-κ*N*) labile interaction between the stable and inert Ru–(PTA-κ*P*) moieties and Zn(TPP). In further agreement with this hypothesis the final spectrum of this series was substantially coincident with that obtained by adding 2 equiv of PTA to a 1:4 mixture of [Ru(TPP)(CO)] and Zn(TPP) (Figures S9 and S10), indicating that PTA discriminates between Ru and Zn even when it is not preventively bound to Ru and is in the presence of a stoichiometric excess of Zn.

**Figure 4:**
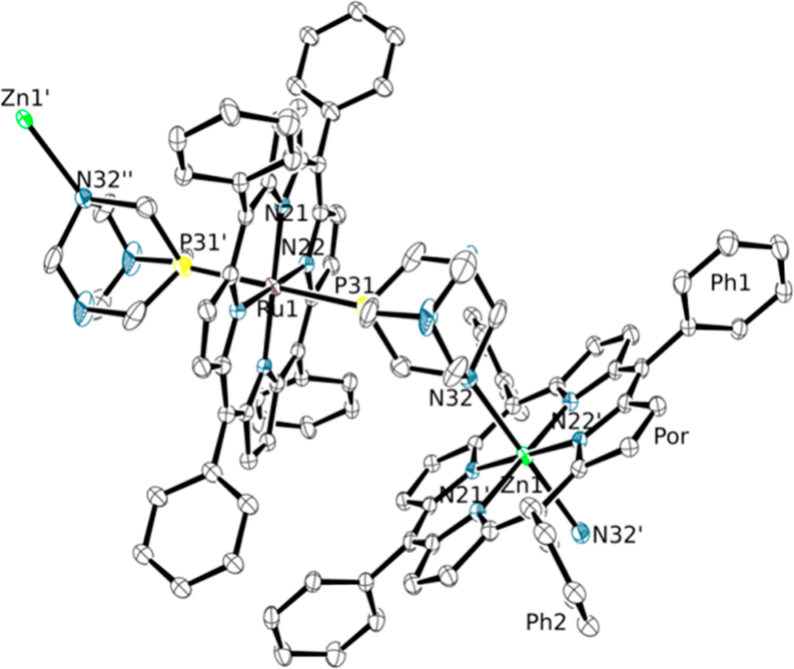
ORTEP representation (50% probability ellipsoids) of the “monomeric” unit of the polymeric zigzag chains which constitute the crystal structure of compound [{Ru(TPP)(PTA-κ^2^*P,N*)_2_}{Zn(TPP)}]_∞_ (**4**) (hydrogen atoms and minor population of one disordered phenyl group omitted for clarity).^47^ The connections of the monomeric unit with the infinite polymeric 1D chain are also evidenced. The Ph1 and Ph2 labels allow one to visualize on the figure the dihedral angles reported in Table S4.

X-ray quality single crystals of the 1D polymeric compound [{Ru(TPP)(PTA-κ^2^*P,N*)_2_}{Zn(TPP)}]_∞_ (**4**) were obtained by slow diffusion of *n*-hexane onto a chloroform solution of a mixture containing 2 equiv of Zn(TPP) per mole of **1**. The crystal structure of compound **4** consists of parallel zigzag polymeric chains, oriented along the crystallographic *c* axis, each formed by a sequence of alternating Ru(TPP) and Zn(TPP) units (Figure 4).

Adjacent Ru/Zn units are connected by a PTA bridging ligand which coordinates to Ru through the phosphorus atom and to Zn through one of the nitrogen atoms (Figures S14–S16). Thus, both Ru and Zn are six-coordinate and feature two equal axial ligands. Each {*trans*-Ru(PTA)_2_} unit binds two zinc atoms with *anti* geometry, thus generating the zigzag motif. Since the equatorial environment of Ru and Zn is identical and the P/N bonding modes of the PTA ligand are nearly geometrically equivalent, the symmetry of the observed diffraction pattern (space group *C*2/*c*) does not distinguish the two metal ions and the corresponding PTA binding modes. This leads to a crystallographically independent fragment in which a single metal site (M) is equally partitioned between Ru and Zn and, correspondingly, two symmetry related binding sites (L) of the PTA are partitioned at 50% between P and N. Consistently, the M–L bond distance of 2.3800(7) Å is intermediate between that of the Ru–(PTA-κ*P*) bond in [Ru(TPP)(PTA-κ*P*)_2_] (**1**, 2.3253(7) Å, see above) and that of the Zn–(PTA-κ*N*) bond in the six-coordinate {Zn(TPP)(PTA-κ^2^*P,N*)_2_} fragment (2.534(2) Å, see below compound **6**). It is to be noted that the Zn–(PTA-κ*N*) bond length is remarkably shorter for five-coordinate [Zn(TPP)(PTA-κ*N*)] (**3**, Table 2). For comparison, the Zn–N bond lengths in similar six-coordinate zinc porphyrin compounds with HTMA, [Zn(TOHPP)(HTMA)_2_] and [Zn(TCPP)(HTMA)_2_] (TOHPP = tetra(4-hydroxyphenyl)porphyrin, TCPP = tetra(4-carboxyphenyl)porphyrin), are remarkably larger than in the five-coordinate [Zn(TPyP)(HTMA)] (TPyP = tetra(4′-pyridyl)porphyrin) (Table 2).^24^ When dissolved in chloroform, compound **4** disassembles into the components, as indicated by the NMR spectra (e.g., the ^31^P NMR spectrum in CDCl_3_ is coincident with that of **1**, see Experimentals).

Next, we addressed the preparation of PTA-bridged Ru/Zn species containing a single metallo-porphyrin, either Ru(TPP) or Zn(TPP), and a complex of the complementary metal, i.e. Zn(II) or Ru(II), respectively. As a first example we choose the symmetrical and coordinatively saturated Ru–PTA complex *cis,cis,trans*-[RuCl_2_(CO)_2_(PTA-κ*P*)_2_] (**5**)^16c^ that features the same {*trans*-Ru(PTA-κ*P*)_2_} fragment as **1**. The results of an NMR titration of Zn(TPP) (from 2 to 4 equiv) into a CDCl_3_ solution of **5** were similar to those described above with **1**, i.e., upfield shift and broadening of the PTA proton resonances (Figure S11). Also in this case, the ^31^P resonance was not particularly affected by the addition of Zn(TPP) and occurred as a singlet at −48.9 ppm (to be compared with −51.0 ppm in the free complex), indicating that the Ru complex remains intact.

**Figure 5:**
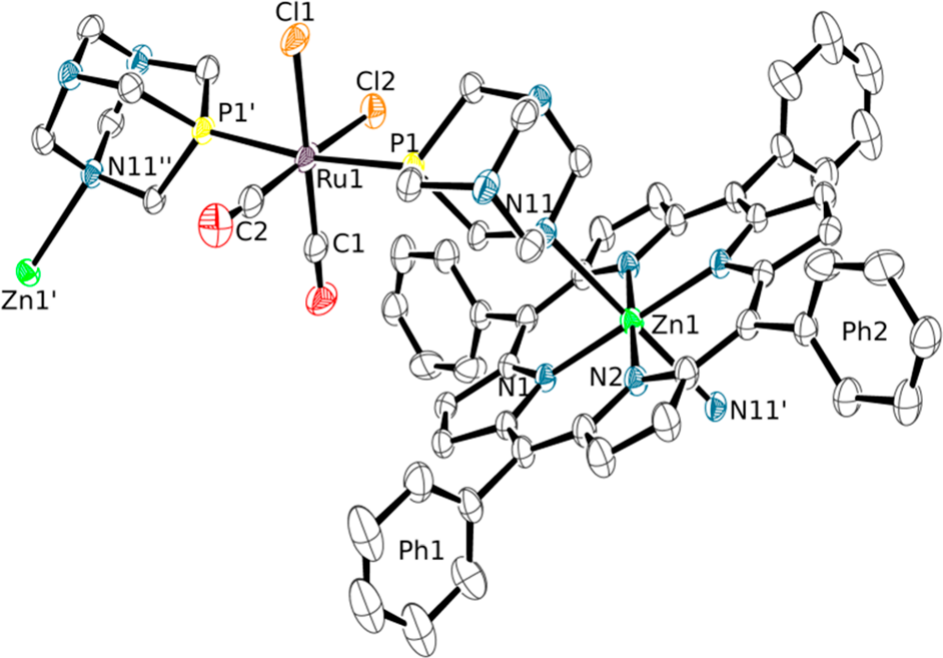
ORTEP representation (50% probability ellipsoids) of the “monomeric” unit of the polymeric Ru–Zn chains which constitute the crystal structure of compound *cis,cis,trans*-[{RuCl_2_(CO)_2_(PTA-κ^2^*P,N*)_2_}{Zn(TPP)}·9.2(H_2_O)]_∞_ (**6**·9.2(H_2_O)). The connections of the monomeric unit with the infinite polymeric 1D chain are also evidenced. Disordered cocrystallized water molecules (that do not interact with the chains) and hydrogen atoms are omitted for clarity. Only half of the Ru and Zn units are crystallographically independent. In the Ru complex, the *trans* C2 carbonyl and Cl2 ligands exchange their positions around a 2-fold axis due to disorder: only one of the two identical populations is represented.^48^ Labels Ph1 and Ph2 allow one to visualize on the figure the dihedral angles reported in Table S5.

**Figure 6:**
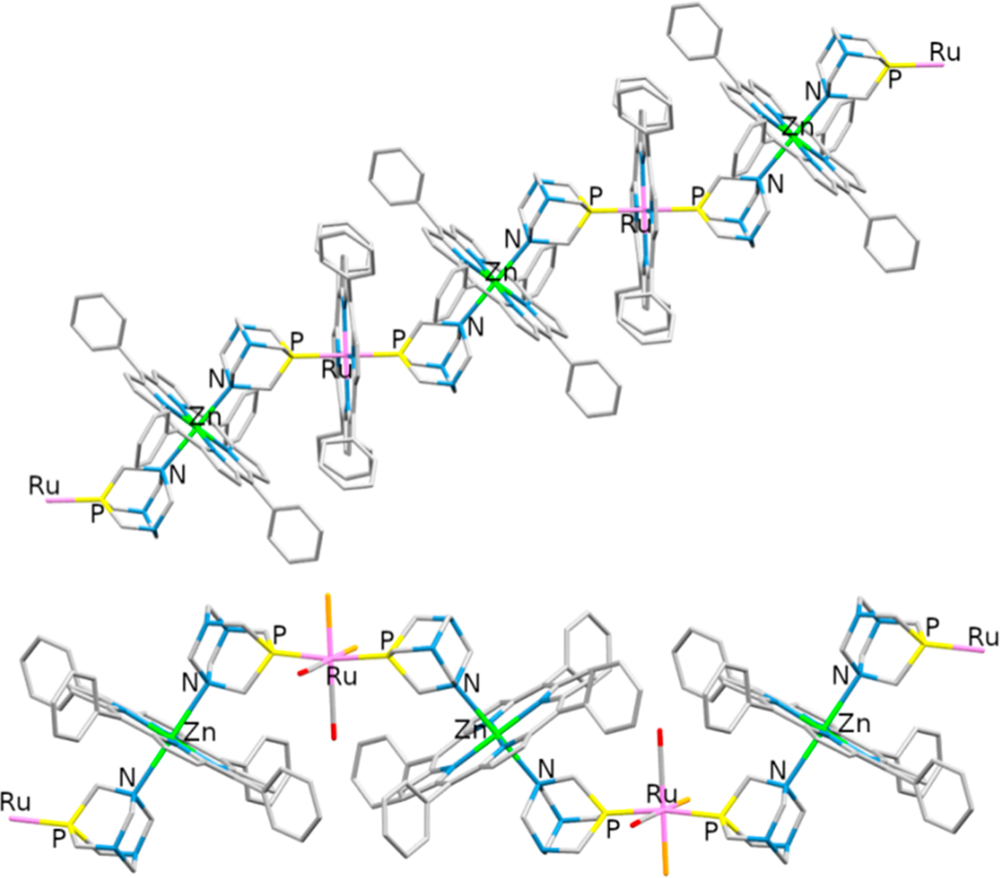
Zigzag and “Greek frame” polymeric chains of [{Ru(TPP)(PTA-κ^2^*P,N*)_2_}{Zn(TPP)}]_∞_ (**4**, top) and *cis,cis,trans*-[{RuCl_2_(CO)_2_(PTA-κ^2^*P,N*)_2_}{Zn(TPP)}·9.2(H_2_O)]_∞_ (**6**·9.2(H_2_O), bottom), respectively, with the {*trans*-Ru(PTA)_2_} fragments iso-oriented (crystallization molecules omitted). Color code: Ru = light purple, Zn = green, *P* = yellow, *N* = blue, O = red, Cl = orange.

X-ray quality single crystals of the 1D polymeric compound *cis,cis,trans*-[{RuCl_2_(CO)_2_(PTA-κ^2^*P,N*)_2_}{Zn(TPP)}]_∞_ (**6**) were obtained upon diffusion of diethyl ether onto a chloroform solution of a 2:1 mixture of Zn(TPP) and **5**. The crystal structure of compound **6** (Figure 5) is similar to that of **4** and consists of parallel polymeric chains in which the Zn atom of each Zn(TPP) is six-coordinate and binds axially two PTA ligands belonging to different Ru complexes. However, since each Ru complex bridges two Zn(TPP) units with *syn* geometry, the resulting chain has “Greek frame” shape (rather than zigzag as in **4**) (Figure S17). The two polymeric chains of **4** and **6** are compared in Figure 6.

**Figure 7:**
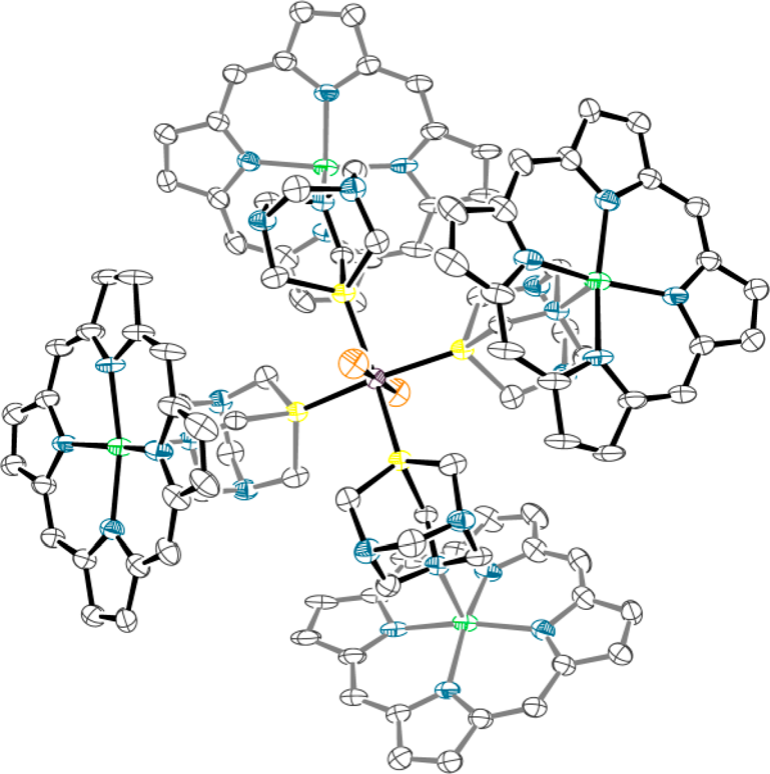
ORTEP representation (50% probability ellipsoids) of the molecule of compound *trans*-[{RuCl_2_(μ-PTA-κ^2^*P,N*)_4_}{Zn(TPP)}_4_]·8/3CHCl_3_·2*n*-hexane (**8**·8/3CHCl_3_·2*n*-hexane) in the crystal structure. The phenyl rings, hydrogen atoms, CHCl_3_, and *n*-hexane solvent molecules are omitted for clarity. The bonds of the two porphyrins that lay below the equatorial plane of the Ru complex and of the two PTA moieties that are partially overlapped by the two upward porphyrins are in gray.

Conversely, the crystallization of Zn(TPP) with another symmetrical Ru–PTA complex, *trans*-[RuCl_2_(PTA-κ*P*)_4_] (**7**),^16,17^ afforded different results, depending on the Zn/PTA ratio adopted. Using a stoichiometric or a slight excess of Zn(TPP) (i.e., Zn/PTA = 1 or 1.5) the discrete molecular species *trans*-[{RuCl_2_(PTA-κ^2^*P,N*)_4_}{Zn(TPP)}_4_] (**8**, Figure 7 and Figures S19−S21), in which each one of the four coplanar PTA ligands of **7** is axially bound through an N atom to a five-coordinate Zn(TPP), was obtained.

The four porphyrins in **8** lay alternatively above and below the equatorial plane of the Ru complex, generating a very compact arrangement (Figure S21) that closely resembles that of the porphyrin pentamer [Zn(3′TPyP){Ru(TPP)(CO)}_4_] (3′TPyP = 5,10,15,20-tetra(3′-pyridyl)porphyrin) described by us 20 years ago.^49^ Similarly to what found for **6**, when redissolved in CDCl_3_ the crystals of **8** give a singlet in the ^31^P{^1^H} NMR spectrum at −51.1 ppm (cfr −50.6 ppm in **7**).

**Figure 8:**
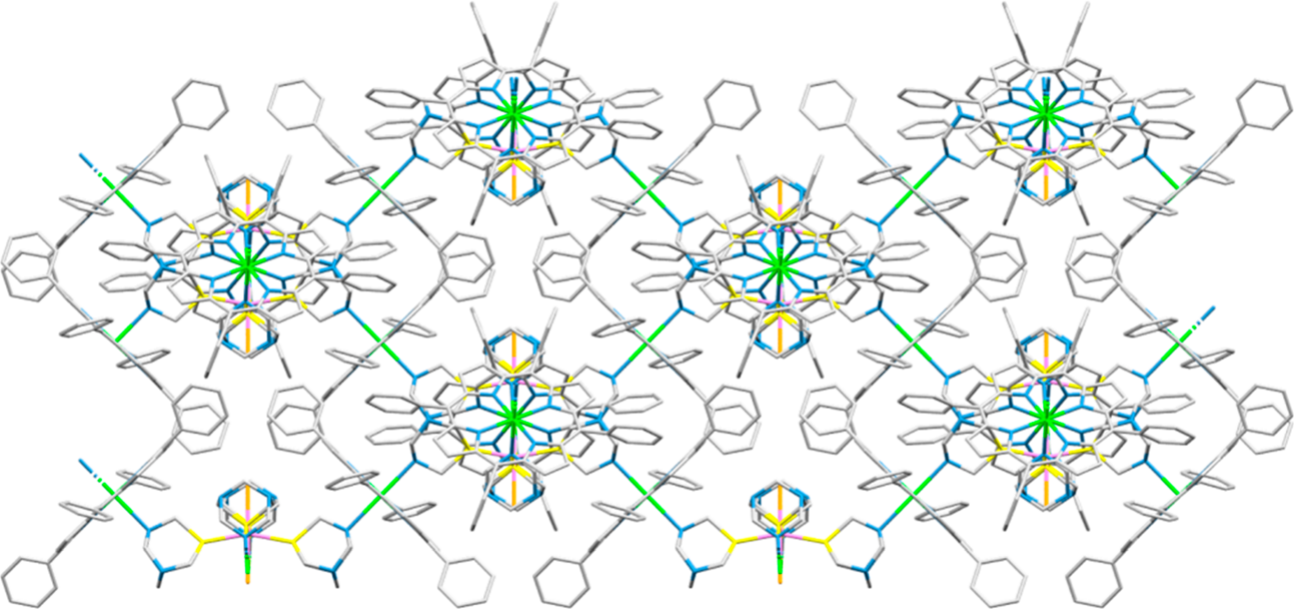
Crystal structure of compound *trans*-[{RuCl_2_(PTA-κ^2^*P,N*)_4_}{Zn(TPP)}_2_]_∞_ (**9**): View along the *b* cell axis of six 1D chains (parallel to *b* and normal to the page), evidencing the parallel and interconnected planes formed by the Ru atoms. The interchain connections are built by PTA–ZnTPP–PTA bridges. When viewed along this direction, the plane of each porphyrin is normal to the page. The Ru atoms along the 1D chains are alternatively connected also to pairs of Ru atoms in adjacent threads that lay in the plane above and below, respectively. Color code: Ru (light purple), Zn (green), P (yellow), N (blue).

**Figure 9:**
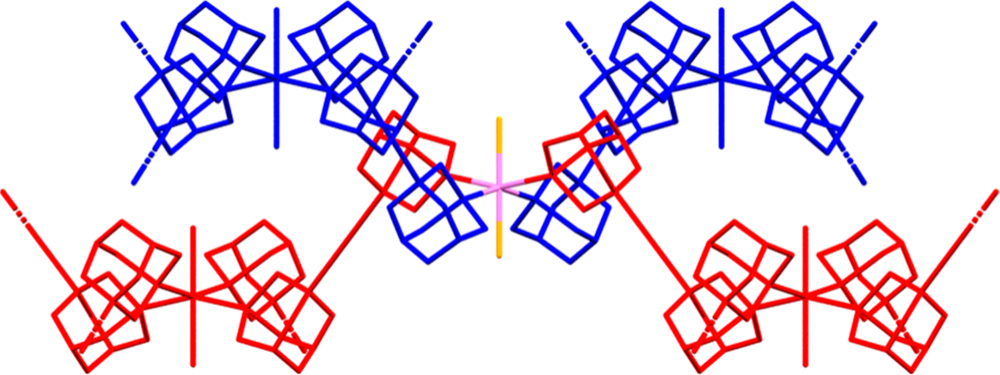
Schematic crystal structure of compound *trans*-[{RuCl_2_(PTA-κ^2^*P,N*)_4_}{Zn(TPP)}_2_]_∞_ (**9**): Perspective view (porphyrins omitted) of two orthogonal {Ru(PTA-κ^2^*P,N*)Zn}_∞_ 1D chains (red and blue) crossing at a Ru center in the crystal structure of complex **9**. The Zn atom is located halfway between the pairs of PTA ligands of any two neighboring Ru moieties.

Conversely, when a defect of Zn(TPP) was used (Zn/PTA = 0.5) crystals of the polymeric network *trans*-[{RuCl_2_(PTA-κ^2^*P,N*)_4_}{Zn(TPP)}_2_]_∞_ (**9**) were obtained. In **9**, each Ru center is surrounded by four Zn(TPP) units with a geometry very similar to that found in **8**. However, in this case the Zn atoms are six-coordinate, thus originating a 3D polymeric network (Figure 8), with a texture of orthogonal 1D threads that intersect each other at every Ru center (Figure 9). Each 1D thread of the network, originated by a {*trans*-Ru(PTA-κ^2^*P,N*)_2_} fragment, has the “Greek frame” shape found in **6**.

Consistent with what observed above (Table 2), the axial Zn–N bond length in six-coordinate **9** (2.4869(2) Å) is similar to that found in **6** (2.534(2) Å), and remarkably longer than that in five-coordinate **8** (2.242(6) Å).

**Figure 10:**
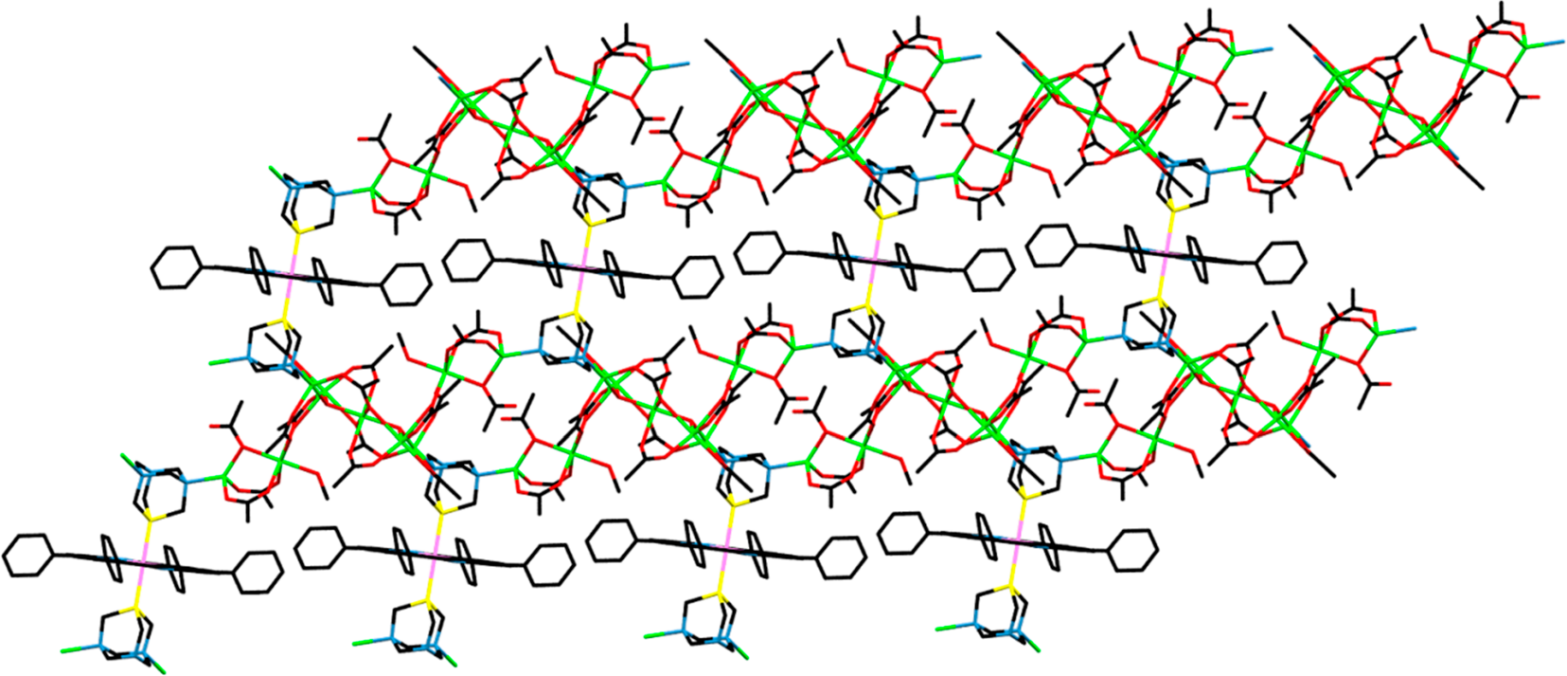
Stick representation of a portion of one of the 2D parallel polymeric layers that constitute the crystal structure of compound [{Ru(TPP)(PTA-κ^3^*P,2N*)_2_}{Zn_9_(CH_3_COO)_16_(CH_3_OH)_2_(OH)_2_}·3CHCl_3_]_∞_ (**10**·3CHCl_3_). Color code: Ru (light purple), Zn (green), P (yellow), N (blue), O (red).

**Figure 11:**
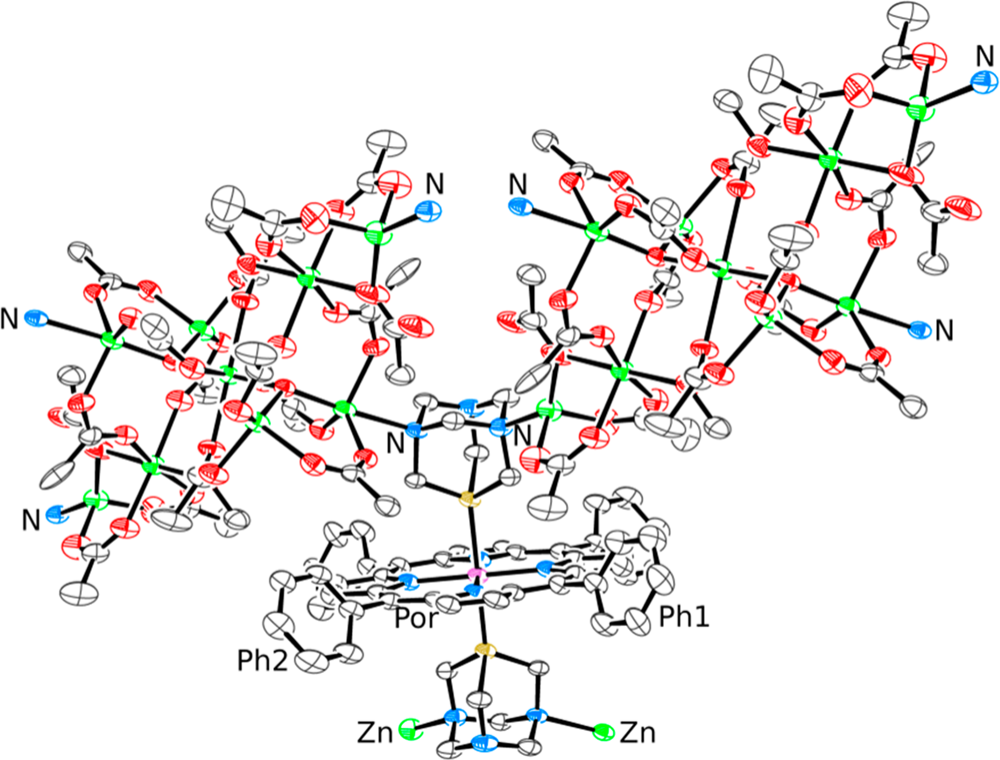
ORTEP representation of a fragment of the structure of compound [{Ru(TPP)(PTA-κ^3^*P,2N*)_2_}{Zn_9_(CH_3_COO)_16_(CH_3_OH)_2_(OH)_2_}·3CHCl_3_]_∞_ (**10**·3CHCl_3_) showing one {Ru(TPP)(PTA-κ^3^*P,2N*)_2_} unit with two polynuclear Zn moieties, each one connected to two N atoms of one of the PTA axial ligands. The two N–Zn bonds connecting the second (lower) PTA ligand to corresponding Zn clusters are also shown, together with the four connections of each Zn cluster with the N atoms of PTA ligands belonging to four distinct [{Ru(TPP)(PTA-κ^3^*P,2N*)_2_} units. Labels Ph1 and Ph2 allow one to visualize on the figure the dihedral angles reported in Table S8. Color code: Ru (light purple), Zn (green), P (yellow), N (blue), O (red).

The crystallization of [Ru(TPP)(PTA-κ*P*)_2_] (**1**) with a ca. 8:1 excess of Zn(CH_3_COO)_2_ afforded crystals of [{Ru(TPP)(PTA-κ^3^*P,2N*)_2_}{Zn_9_(CH_3_COO)_16_(CH_3_OH)_2_(OH)_2_}·3CHCl_3_]_∞_ (**10**·3CHCl_3_). The crystal structure of compound **10** (Figure 10) can be described as a stack of 2D polymeric layers, almost perfectly parallel to the plane defined by the *b* axis and the diagonal of the ac face of the unit cell. Each polymeric layer contains the Ru porphyrin and an intricate neutral Zn–acetate cluster in 1:1 ratio (for the description of the Zn_9_ cluster see the Supporting Information). The Ru and the central Zn atom (Zn4) sit on inversion points, so that only half of the Ru porphyrin and Zn cluster are crystallographically independent. Four Zn atoms of each cluster are N-bound to four PTA ligands of different Ru(TPP) units, and correspondingly, each Ru porphyrin connects with four Zn clusters, two for each axial PTA ligand (Figure 11). Thus, in this case PTA has a triple-bridging κ^3^*P,2N* binding mode.

Due to the layered structure, the unit cell contains a cavity whose volume amounts to 16% of the total (see Experimental Section).

**Figure 12:**
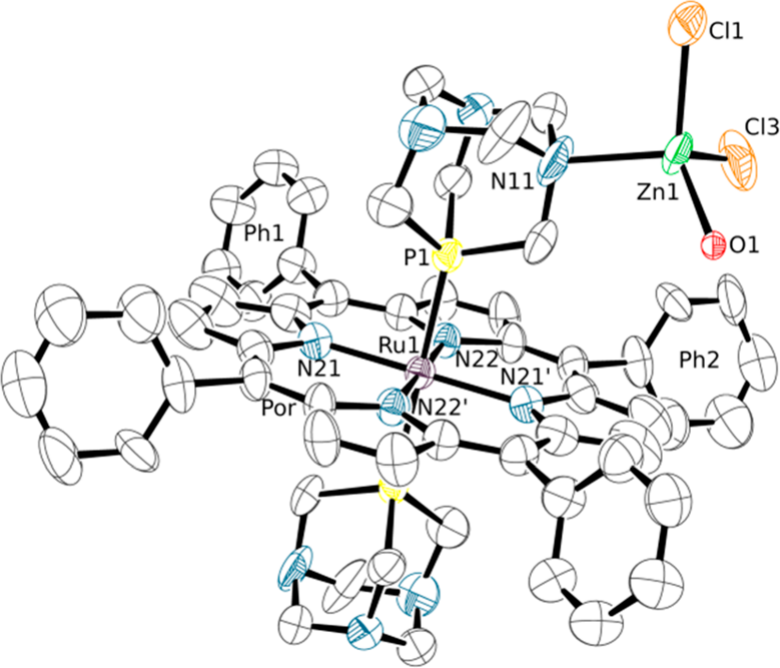
ORTEP representation (50% probability ellipsoids) of compound [{Ru(TPP)(PTA-κ*P*) (PTA-κ^2^*P,N*)}{ZnCl_2_(OH_2_)}·0.6CHCl_3_] (**11**·0.6CHCl_3_). Only the major population (SOF = 0.3) of the disordered {ZnCl_2_(OH_2_)} group has been represented. Primed labels indicate symmetry mates. Hydrogen atoms and a disordered CHCl_3_ crystallization molecule are omitted for clarity. Labels Ph1 and Ph2 allow one to visualize on the figure the dihedral angles reported in Table S9. Color code: Ru (light purple), Zn (green), P (yellow), N (blue), Cl (orange).

By changing the nature of the zinc salt a remarkably different compound was obtained. In fact, diffusion of *n*-hexane into a chloroform/ethanol solution of a 1:2 mixture of **1** with ZnCl_2_ afforded crystals of the dinuclear compound [{Ru(TPP)(PTA-κ*P*)(PTA-κ^2^*P,N*)}{ZnCl_2_(OH_2_)}] (**11**) in which one of the two *trans* PTA-κ*P* ligands of **1** binds through an N atom to a {ZnCl_2_(OH_2_)} fragment (Figure 12). The distorted tetrahedral coordination environment of the Zn atom is similar to that found in [ZnCl_2_(OH_2_)(PTA=O)].^46^ The crystal structure consists of an arrangement of parallel 1D sequences of molecules of complex **11**, oriented along the [101] direction, with a shape that closely resembles the “Greek frame” found in **6** and **9** (Figure S25). Regretfully, due to the low quality of the X-ray data (see also the Experimental Section for details) the expected slight elongation of the PTA C–N11(Zn) bond distances could not be detected.^50^

## Conclusions

In this work, we demonstrated that PTA (1,3,5-triaza-7-phosphaadamantane) behaves as an orthogonal ligand between Ru(II) and Zn(II), since it selectively binds through the P atom to ruthenium and through one or more of the N atoms to zinc. This property of PTA was first exploited by us for preparing the two monomeric porphyrin adducts [Ru(TPP)(PTA-κ*P*)_2_] (**1**) and [Zn(TPP)(PTA-κ*N*)] (**3**), in which PTA is axially bound to the inner metal. Then, we prepared a number of heterobimetallic Ru/Zn porphyrin polymeric networks–and two discrete species–mediated by *P,N*-bridging PTA in which either both metals reside inside a porphyrin core, or one metal belongs to a porphyrin, either Ru(TPP) or Zn(TPP), and the other to a complex or salt of the complementary metal (i.e., *cis,cis,trans*-[RuCl_2_(CO)_2_(PTA-κ*P*)_2_] (**5**), *trans*-[RuCl_2_(PTA-κ*P*)_4_] (**7**), Zn(CH_3_COO)_2_, and ZnCl_2_). Both the molecular compounds **1**, **3**, *trans*-[RuCl_2_(PTA-κ^2^*P,N*)_4_}{Zn(TPP)}_4_] (**8**), and [{Ru(TPP)(PTA-κ*P*)(PTA-κ^2^*P,N*)}{ZnCl_2_(OH_2_)}] (**11**), and the polymeric species [{Ru(TPP)(PTA-κ^2^*P,N*)_2_}{Zn(TPP)}]_∞_ (**4**), *cis,cis,trans*-[{RuCl_2_(CO)_2_(PTA-κ^2^*P,N*)_2_{Zn(TPP)}]_∞_ (**6**), *trans*-[{RuCl_2_(PTA-κ^2^*P,N*)_4_}{Zn(TPP)}_2_]_∞_ (**9**), and [{Ru(TPP)(PTA-κ^3^*P,2N*)_2_}{Zn_9_(CH_3_COO)_16_(CH_3_OH)_2_(OH)_2_}]_∞_ (**10**) have been structurally characterized by single crystal X-ray diffraction. The number of compounds with the relatively rare six-coordinate Zn(TPP) (three, the polymeric networks of **4**, **6**, and **9**, out of five) is largely above-average (see ref (45)), strongly suggesting that the stereoelectronic features of PTA are particularly well-suited for this type of coordination. In **4**, **6**, **8**, **9**, and **11** the bridging PTA has the κ^2^*P,N* binding mode, whereas in the 2D polymeric layers of **10** it has the triple-bridging mode κ^3^*P*,2*N*. In one case, we demonstrated that, by tuning the PTA/Zn(TPP) ratio, it is possible to control the number of axial Zn–N coordination bonds and thus to switch from a molecular species (**8**, five-coordinate Zn) to a 2D polymeric network (**9**, six-coordinate Zn). Similarly, we are confident that also in the case of **11** by operating at higher Ru/ZnCl_2_ ratios a second PTA ligand (from a different **1**) is likely to replace the residual water molecule on the Zn fragment, thus affording a polymeric network upon crystallization. Interestingly, we also found that when Zn(TPP) is sandwiched between two {*trans*-Ru(PTA-κ^2^*P,N*)_2_} fragments, similar 1D polymeric chains with two different shapes—zigzag in **4** vs “Greek frame” in **6** and **9**—are obtained depending on whether the connecting bonds of each Ru fragment have an *anti* (**4**) or *syn* geometry (**6** and **9**).

Due to the rather weak and labile nature of the Zn–N(PTA) bond, and consistent with literature data about Zn–PTA adducts,^14,15,46^ the compounds are not stable in solution and disassemble into the mononuclear fragments in concentration-dependent equilibria. Thus, their main interest resides in solid-state features. Porphyrin MOFs^51^—of which compounds **4**, **6**, **9**, and **10** are the first examples mediated by PTA—are extensively investigated in several fields, such as light-harvesting, guest inclusion, photodynamic therapy, and (photo)catalysis.^52^

We believe that the examples reported in this work represent robust proofs-of-concept that firmly establish the binding preferences of PTA toward Ru(II) and Zn(II), and are confident that a variety of discrete species and networks can be produced by changing the nature of the Ru and Zn partners and their ratio. In particular, there are several inert Ru(II) compounds (in addition to **5** and **7**) that feature two or more P-bonded PTA ligands that might be exploited as linkers of well-defined geometry for the rational design of solid state networks with Zn–porphyrins (or other Zn compounds). The remaining ancillary ligands on the Ru center would allow to fine-tune the properties of the network, e.g., by providing interactions for the selective binding of host molecules. Finally, the uncoordinated N atoms of PTA in the networks might undergo protonation, thus introducing positive charges and the possibility of making additional electrostatic and H-bonding interactions.

## Experimental Section

### Materials

All chemicals, including TLC silica gel plates, were purchased from Sigma-Aldrich and used as received. Solvents were of reagent grade. The ruthenium precursor *cis,cis,trans*-[RuCl_2_(CO)_2_(PTA-κ*P*)_2_] (**5**),^16c^*trans*-[RuCl_2_(PTA-κ*P*)_4_] (**7**),^16a,17^ and the porphyrins TPP,^53^ [Ru(TPP)(CO)],^54^ and Zn(TPP) were synthesized and purified as previously reported by us or by others.^55^

### Instrumental Methods

Mono- and bidimensional (^1^H–^1^H COSY, ^1^H–^13^C HSQC) NMR spectra were recorded at room temperature on a Varian 400 or 500 spectrometer (^1^H: 400 or 500 MHz, ^31^P{^1^H}: 161 or 202 MHz). ^1^H chemical shifts in CDCl_3_ were referenced to the peak of residual nondeuterated solvent (δ = 7.26). ^31^P{^1^H} chemical shifts were measured relative to external 85% H_3_PO_4_ at 0.00 ppm. ESI mass spectra were collected in the positive mode on a PerkinElmer APII spectrometer at 5600 eV. The UV–vis spectra were obtained on an Agilent Cary 60 spectrophotometer, using 1.0 cm path-length quartz cuvettes (3.0 mL). Chloroform spectra in the CO stretching region were recorded between CaF_2_ windows (0.5 mm spacer) on a PerkinElmer Fourier-transform IR/Raman 2000 instrument in the transmission mode. Elemental analyses were performed on a Thermo Flash 2000 CHNS/O analyzer in the Department of Chemistry of the University of Bologna (Italy).

### X-ray Diffraction

Data collections were performed at the X-ray diffraction beamline (XRD1) of the Elettra Synchrotron of Trieste (Italy) equipped with a Pilatus 2 M image plate detector.

Collection temperature was 100 K (nitrogen stream supplied through an Oxford Cryostream 700); the wavelength of the monochromatic X-ray beam was 0.700 Å and the diffractograms were obtained with the rotating crystal method. The crystals were dipped in *N*-paratone and mounted on the goniometer head with a nylon loop. The diffraction data were indexed, integrated and scaled using the XDS code.^56^ The structures were solved by the dual space algorithm implemented in the SHELXT code.^57^ Fourier analysis and refinement were performed by the full-matrix least-squares methods based on *F*^2^ implemented in SHELXL.^58^ The Coot program was used for modeling.^59^ Anisotropic thermal motion was allowed for all non-hydrogen atoms. Hydrogen atoms were placed at calculated positions with isotropic factors *U* = 1.2 × *U*_eq_, where *U*_eq_ is the equivalent isotropic thermal factor of the bonded non hydrogen atom. Crystal data and details of refinements are given in the Supporting Information.

In the case of compound **6**, an initial refinement of the structure afforded an R value of 12.78% and two Fourier peaks at unreasonable positions; a first intense peak was found at about 0.90 Å from the Ru atom, while a second less intense peak was located between the N atoms of two PTA ligands of adjacent chains, making completely unreasonable bonding angles. This made us suspect the presence of a lattice translocation defect (LTD).^60^ This suspicion was reinforced by the inspection of the diffractograms, where alternating rows of well-defined and streaky spots were apparent. A translocation vector (0,^1^/_2_,^1^/_2_) could be found by assuming that the intense peak near the Ru site was due to another Ru atom of the translocated lattice and by matching the distance of the LTD peak from the Ru site (at (^1^/_2_,0.79,^1^/_2_)). The measured reflection intensities were then corrected according to eq 3 in ref (60). An optimal value of 0.094 for the translocated cell fraction could be found by trial and error until the intensities of the two peaks due to the LTD were reduced to negligible values.

For compound **9**, no Fourier peaks of appreciable intensity could be located inside the mentioned cavity, which was then assumed to contain heavily disordered methanol solvent molecules and modeled with the Squeeze procedure of the PLATON code.^61^ The “squeezed” electronic charge was 148 (in electron charge units), corresponding to about eight methanol solvent molecules.

In the asymmetric unit of the crystal structure of complex **11**, the {ZnCl_2_(OH_2_)} group is very close to a 2-fold axis, thus ruling out the possibility that it is present on both PTA ligands of the *same* Ru complex: in this case, two {ZnCl_2_(OH_2_)} groups of adjacent Ru complexes would overlap (Figure S25). For this reason, we refined a model in which the {ZnCl_2_(OH_2_)} has a total occupation factor of 0.5, which ensures a Ru:Zn ratio of 1:1 (also the Ru atom sits on a special position with occupation factor 0.5). An additional complication is that the {ZnCl_2_(OH_2_)} group is disordered over two positions, which led to a further partition of the 0.5 occupation factor into two populations with occupations of 0.3 and 0.2, respectively (Figure S26).

### Synthesis of the Complexes

#### [Ru(TPP)(PTA-κ*P*)_2_] (**1**)

A 20 mg amount of [Ru(TPP)(CO)] (0.027 mmol) was dissolved in 8 mL of chloroform, obtaining a clear red-purple solution. Upon addition of 2.4 equiv of PTA (10 mg) the solution became immediately darker. The solution was concentrated to ca. 2 mL. Slow evaporation of the solvent afforded within a few hours X-ray quality purple crystals of [Ru(TPP)(PTA-κ*P*)_2_]· 2CHCl_3_ that were filtered after 24h, washed with diethyl ether and dried *in vacuo.* (Yield 25.0 mg, 90%). Anal. Calcd for [C_56_H_52_N_10_P_2_Ru]·2(CHCl_3_) (*M*_w_: 1266.9): C 54.99; H 4.30; N 11.06. Found: C 54.90; H 4.36; N 11.12. ^1^H NMR (CDCl_3_), δ (ppm): 8.34 (s, 8H, β), 8.06 (m, 8H, *o*), 7.67 (m, 12H, *m* + *p*), 3.20 (d, *J* = 12.9 Hz, 6H NC*H*_2_N), 2.55 (d, *J* = 12.9 Hz, 6H NC*H*_2_N), – 0.26 (br s, 12H NC*H*_2_P). Selected ^13^C NMR signals (from the HSQC spectrum) in CDCl_3_, δ (ppm): 133.8 (*o*), 131.9 (β), 126.8 (*m* + *p*), 71.3 (N*C*H_2_N), 45.0 (N*C*H_2_P)_._^31^P{^1^H} NMR (CDCl_3_), δ (ppm): – 50.6 (s, 2P, mutually *trans* PTAs). ESI mass spectrum (*m*/*z*): 1020.1 ([M + H]^+^), 874.1 ([M–PTA+H]^+^), 718.1 ([M–2PTA+H]^+^). UV–vis (CHCl_3_): λ_max_ (ε, L mol^–1^ cm^–1^) = 431 (125400), 523 (8300), 554 (5400) nm.

#### [Ru(TPP)(CO)(PTA-κ*P*)] (**2**)

As noted in the text, this elusive intermediate was observed during the titration of [Ru(TPP)(CO)] with PTA but could not be isolated. ^1^H NMR (CDCl_3_), δ (ppm): 8.62 (s, 8H, β), 8.24, 8.02 (m, 8H, *o + o′*), 7.68 (m, 12H, *m* + *m′* + *p*), 3.11 (d, *J* = 13.0 Hz, 3H NC*H*_2_N), 2.39 (d, *J* = 13.0 Hz, 3H NC*H*_2_N), – 0.71 (br s, 6H NC*H*_2_P). ^31^P{^1^H} NMR (CDCl_3_), δ (ppm): – 60.5 (s, 1P, PTA *trans* CO). Selected IR absorption (chloroform solution, cm^–1^): 1989 (ν_CO_).

The following preparations were performed on a small scale (maximum 5–6 mg of the limiting reagent) with the specific aim of obtaining X-ray quality single crystals by slow diffusion of a precipitating solvent into ca. millimolar solutions of the reagents in the indicated molar ratios. Yields were not measured. The ^1^H NMR spectra of the adducts are not reported, since—due to the labile nature of the Zn–N bonds—they depend on the concentration. In the NMR titrations, the metalloporphyrin concentration was ca. 5 mM.

#### [Zn(TPP)(PTA-κ*N*)]·H_2_O·CHCl_3_ (**3**·H_2_O·CHCl_3_)

Crystals of **3**·H_2_O·CHCl_3_ were obtained by slow diffusion of diethyl ether into a ca. 5 mM chloroform solution of a 2:1 Zn(TPP)/PTA mixture. ^31^P{^1^H} NMR (CDCl_3_) δ (ppm): −102.1 (s).

#### [{Ru(TPP)(PTA-κ^2^*P,N*)_2_}{Zn(TPP)}]_∞_ (**4**)

X-ray quality crystals of **4** were obtained upon diffusion of *n*-hexane into a chloroform solution of a ca. 3 mM 2:1 mixture of Zn(TPP) and **1**. ^31^P{^1^H} NMR (CDCl_3_) δ (ppm): −50.1 (s).

#### *cis,cis,trans*-[{RuCl_2_(CO)_2_(PTA-κ^2^*P,N*)_2_}{Zn(TPP)}·9.2(H_2_O)]_∞_ (**6**·9.2(H_2_O))

X-ray quality crystals of **6**·9.2(H_2_O) were obtained upon diffusion of diethyl ether into a chloroform solution of a 1:2 mixture of *cis,cis,trans-*[RuCl_2_(CO)_2_(PTA-κ*P*)_2_] (**5**, 6.1 mg, 0.011 mmol) and Zn(TPP) (2.5 mg, 2 equiv). ^31^P{^1^H} NMR (CDCl_3_) δ (ppm): −49.0 (s).

#### *trans*-[{RuCl_2_(PTA-κ^2^*P,N*)_4_}{Zn(TPP)}_4_]·^8^/_3_CHCl_3_·2*n*-hexane (**8**·^8^/_3_CHCl_3_·2*n*-hexane)

X-ray quality crystals of **8**·8/3CHCl_3_·2C_6_H_14_ were obtained upon diffusion of *n-*hexane into 5 mL of a 1:4 chloroform solution of *trans-*[RuCl_2_(PTA-κ*P*)_4_] (**7**, 5.3 mg, 0.0062 mmol) and Zn(TPP) (17.9 mg, 4 equiv). ^31^P{^1^H} NMR (CDCl_3_) δ (ppm): −51.1 (s).

#### *trans*-[{RuCl_2_(PTA-κ^2^*P,N*)_4_}{Zn(TPP)}_2_·4CHCl_3_]_∞_ (**9**·4CHCl_3_)

X-ray quality crystals of **9**·4CHCl_3_·were obtained upon diffusion of hexane into 4 mL of a 1:2 chloroform solution of *trans-*[RuCl_2_(PTA-κ*P*)_4_] (**7**, 5.0 mg, 0.0062 mmol) and Zn(TPP) (8.4 mg, 2 equiv). ^31^P{^1^H} NMR (CDCl_3_) δ (ppm): −50.7 (s).

#### [{Ru(TPP)(PTA-κ^3^*P,2N*)_2_}{Zn_9_(CH_3_COO)_16_(CH_3_OH)_2_(OH)_2_}·3CHCl_3_]_**∞**_ (**10**·3CHCl_3_)

X-ray quality crystals of **10**·3(CHCl_3_) were obtained by slow diffusion of *n*-hexane into 3 mL of a 5:1 chloroform/methanol solution of a ca. 8:1 mixture of Zn(CH_3_COO)_2_ and [Ru(TPP)(PTA-κ*P*)_2_] (**1**, 3 mg, 0.0023 mmol).

#### [{Ru(TPP)(PTA-κ*P*)(PTA-κ^**2**^*P,N*)}{ZnCl_2_(OH_2_)}·0.6CHCl_3_] (**11**·0.6CHCl_3_)

X-ray quality crystals of **11**·0.6CHCl_3_ were obtained by slow diffusion of *n*-hexane onto 2.4 mL of a 5:1 chloroform/ethanol solution of a 1:2 mixture of [Ru(TPP)(PTA-κ*P*)_2_] (**1**, 3 mg, 0.0023 mmol) and ZnCl_2_.

## Supporting Information Available

Details of X-ray data collection and refinement for compounds **1**, **3**, **4**, **6**, **8**–**11**, mono- and bidimensional NMR spectra of the reported compounds, additional drawings for the X-ray structures, a description of the Zn–acetate cluster of compound **10**, and additional comments on the X-ray structure of compound **11** (PDF)

## Accession Codes

CCDC 1964277, 1964280–1964285, and 1964342 contain the supplementary crystallographic data for this paper. These data can be obtained free of charge via www.ccdc.cam.ac.uk/data_request/cif, or by emailing data_request@ccdc.cam.ac.uk, or by contacting The Cambridge Crystallographic Data Centre, 12 Union Road, Cambridge CB2 1EZ, UK; fax: +44 1223 336033.
