# Supplementary material for: Orthogonal Coordination Chemistry of PTA toward Ru(II) and Zn(II) (PTA = 1,3,5-Triaza-7-phosphaadamantane) for the Construction of 1D and 2D Metal-Mediated Porphyrin Networks
Source: Inorg Chem. 2020 Feb 26;59(6):4068–79. doi: 10.1021/acs.inorgchem.0c00080 (PMC7997375; doi:10.1021/acs.inorgchem.0c00080)
Supplement: Supplementary file 1 — ic0c00080_si_001.pdf [file ic0c00080_si_001.pdf]

The orthogonal coordination chemistry of PTA towards Ru(II) and Zn(II) (PTA = 1,3,5-triaza-7-phosphaadamantane) for the construction of 1D and 2D metal-mediated porphyrin networks.

Federica Battistin,[a†] Alessio Vidal,[a] Paolo Cavigli,[a‡] Gabriele Balducci,[a]* Elisabetta Iengo,[a] Enzo Alessio[a]*

[a] Department of Chemical and Pharmaceutical Sciences, University of Trieste, Via L. Giorgieri 1, 34127 Trieste, Italy. Email: balducci@units.it; alessi@units.it

[†] now at IMDEA Nanociencia, Faraday 9, Ciudad Universitaria de Cantoblanco, 28049 Madrid, Spain.

[‡] now at Department of Agricultural, Food, Enviromental and Animal Sciences, University of Udine, Via Cotonificio 108, 33100 Udine, Italy.

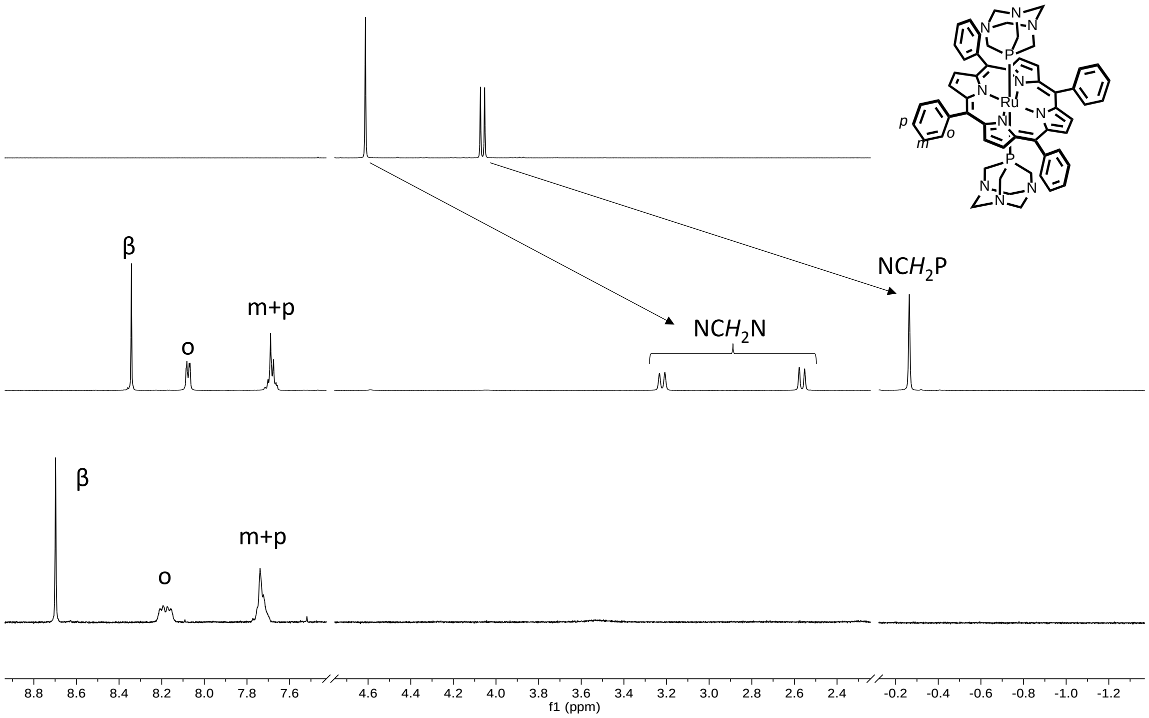

Figure S1. ^{1}H NMR spectra of free PTA (top), [Ru(TPP)(PTA-κ*P*)$_2$] (**1**) (middle) and [Ru(TPP)(CO)] (bottom) in $CDCl_3$.

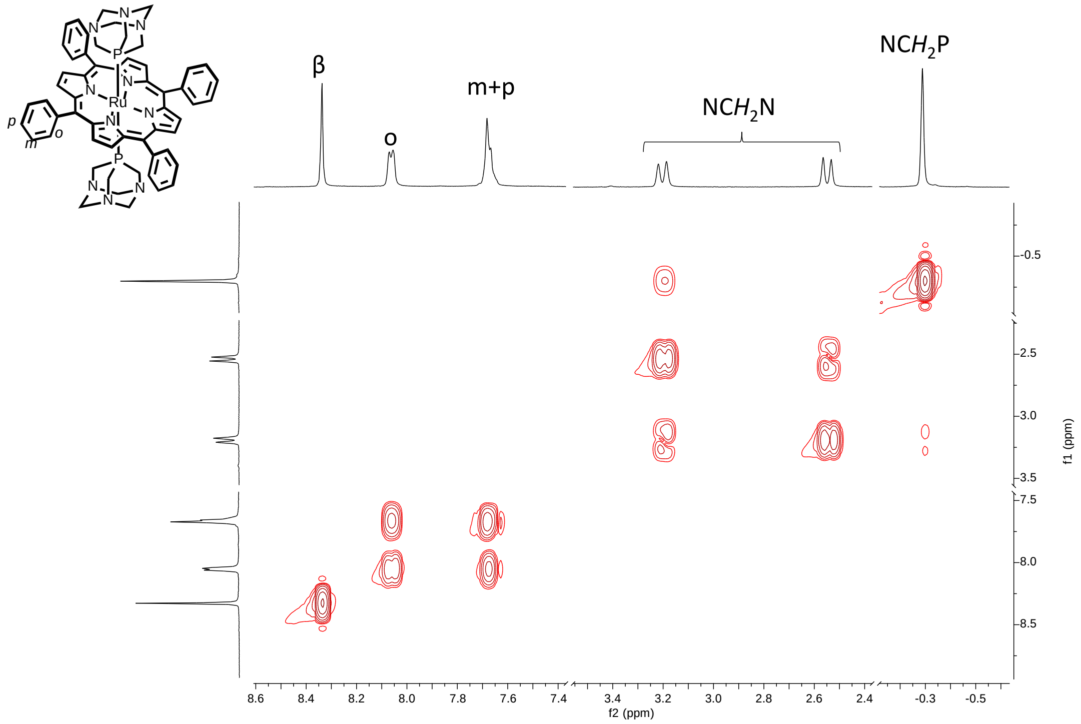

Figure S2. ^{1}H-^{1}H COSY NMR spectrum of [Ru(TPP)(PTA-κ*P*)$_2$] (**1**) in $CDCl_3$.

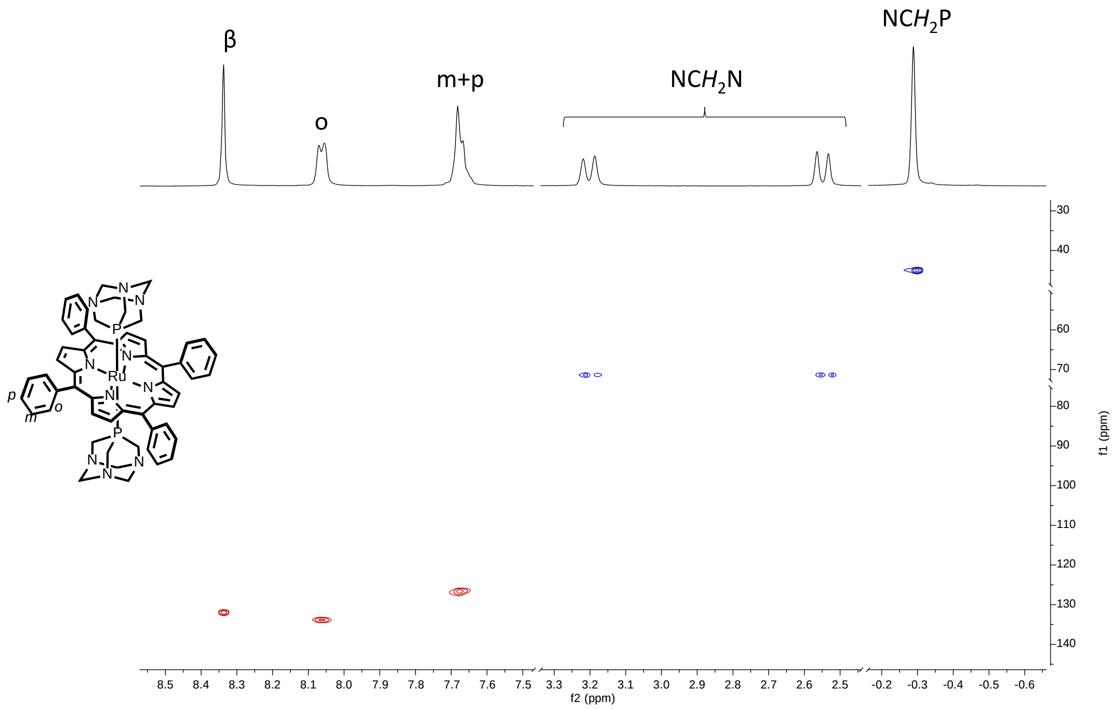

Figure S3. ^{1}H-^{13}C HSQC NMR spectrum of [Ru(TPP)(PTA-κ*P*)$_2$] (**1**) in $CDCl_3$.

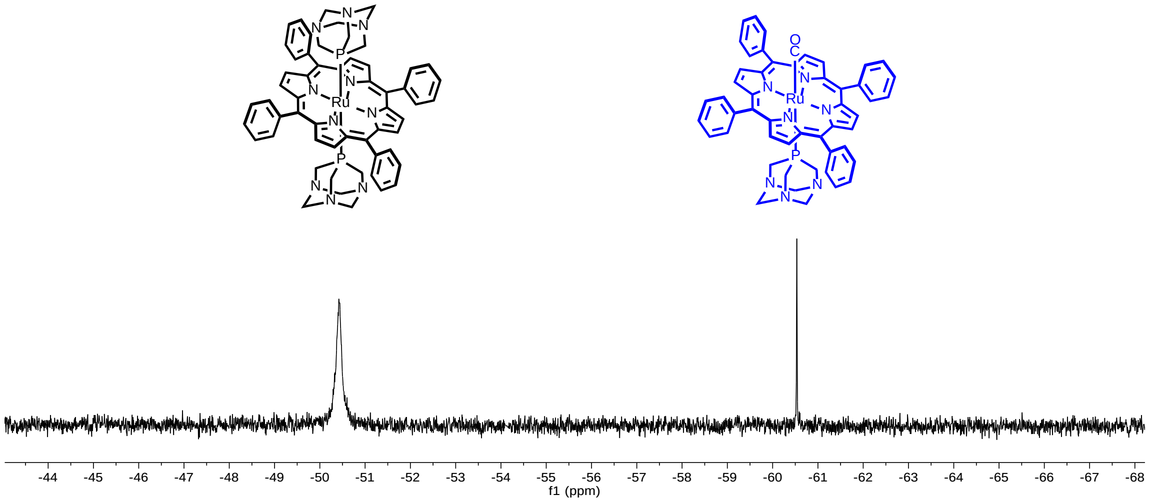

Figure S4. $^{31}P\{^{1}H\}$ NMR spectrum of a mixture of [Ru(TPP)(PTA-κ*P*)$_2$] (**1**) and [Ru(TPP)(CO)(PTA-κ*P*)] (**2**) $CDCl_3$ obtained after the addition of ca. 1 equiv of PTA into a $CDCl_3$ solution of [Ru(TPP)(CO)].

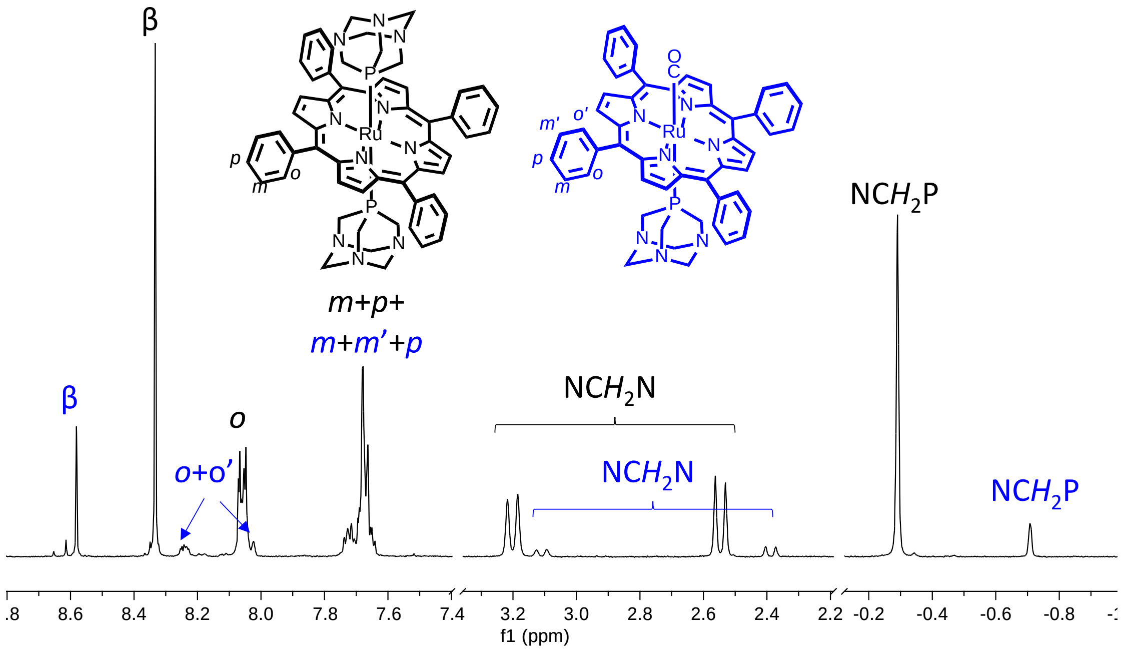

Figure S5. ^{1}H NMR spectrum ($CDCl_3$) of a ca. 3:1 mixture of [Ru(TPP)(PTA-κ*P*)$_2$] (**1**, black labels) and [Ru(TPP)(CO)(PTA-κ*P*)] (**2**, blue labels) obtained during a titration of a [Ru(TPP)(CO)] solution with PTA.

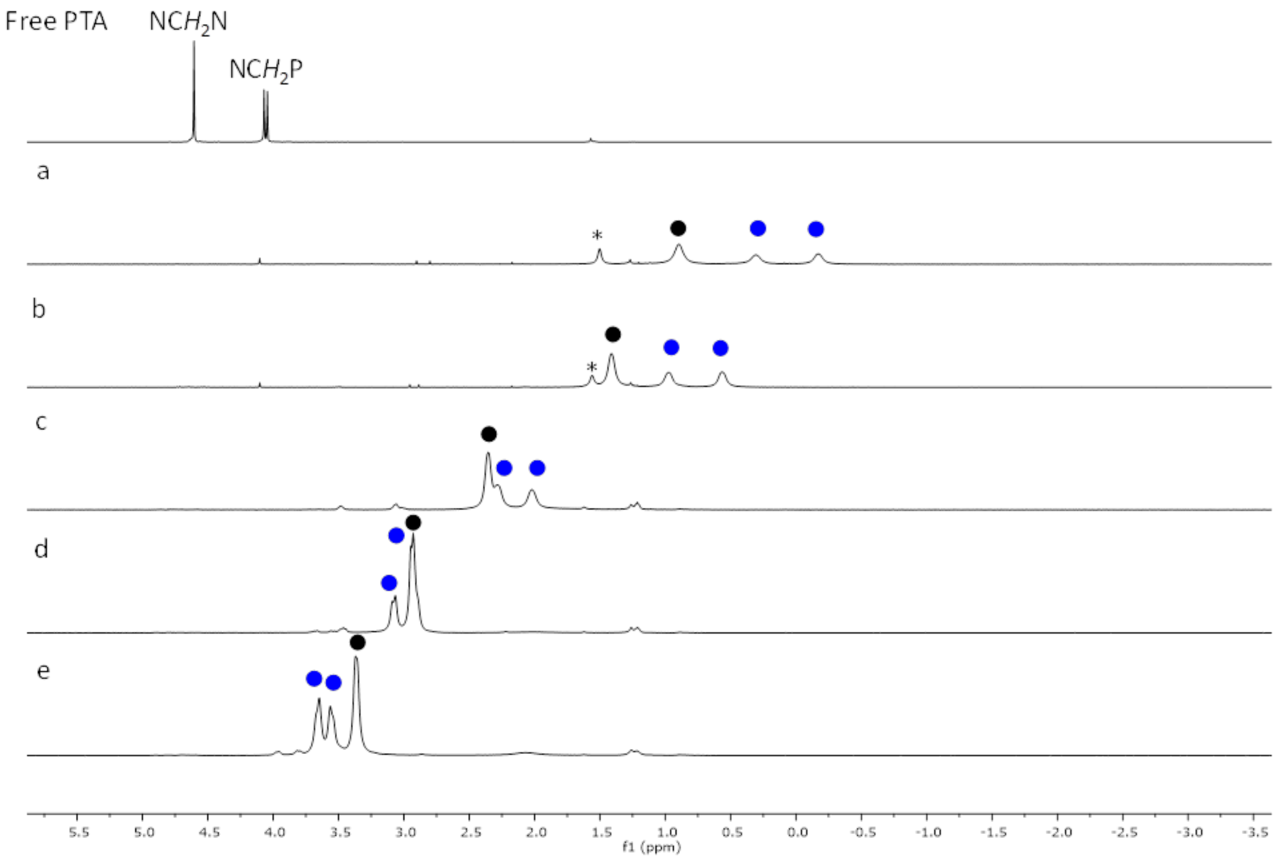

Figure S6. ^{1}H NMR titration of Zn(TPP) with 0.5 (a), 1 (b), 2 (c), 3 (d) and 5 (e) equiv of PTA in $CDCl_3$. The resonance of the NCH_2N protons is labeled with blue dots, and that of the NCH_2P protons with black dots. In the more magnified spectra (a) and (b) the peak of residual water is labeled with *. The porphyrin resonances are not shown.

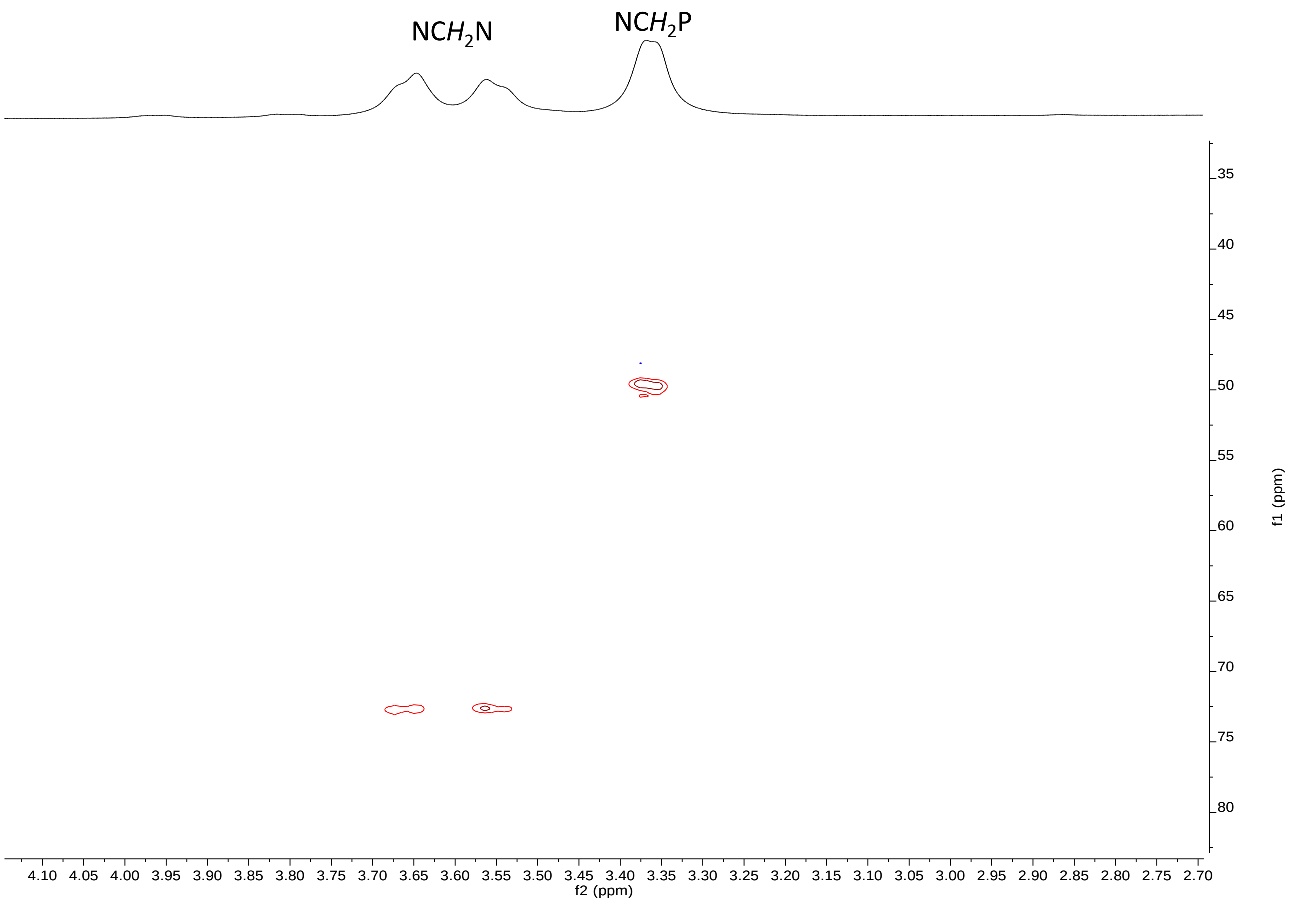

Figure S7. PTA region of the ^{1}H-^{13}C HSQC NMR spectrum of the 1:5 mixture of Zn(TPP) and PTA in $CDCl_3$ (i.e. of spectrum (e) in Figure S6).

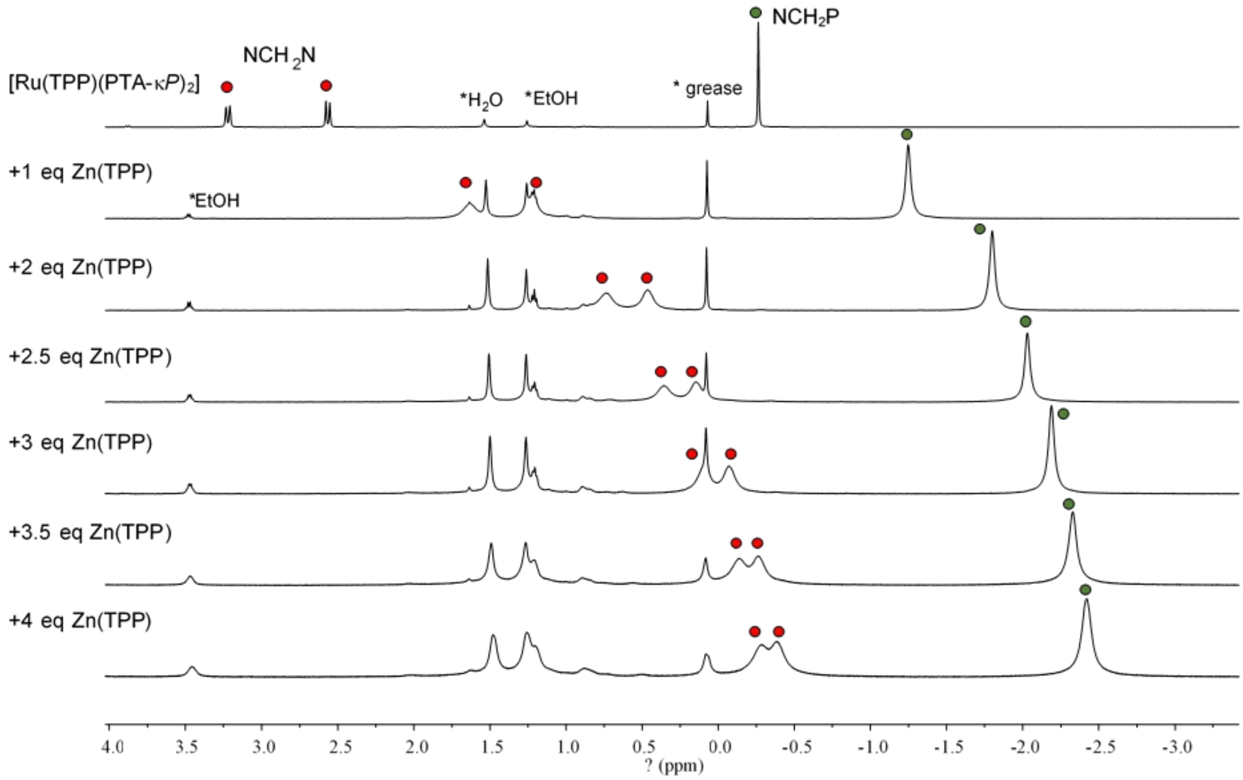

Figure S8. Region of the PTA resonances (red and green dots) in ^{1}H NMR titration of [Ru(TPP)(PTA-κ*P*)$_2$] (**1**) with Zn(TPP) (from 1 to 4 equiv) in $CDCl_3$. The additional resonances in the upfield region, that do not shift in the titration but become broader, belong to grease, H_2O and EtOH. This latter increases since it derives also from Zn(TPP), that should be formulated as [Zn(TPP)(EtOH)].

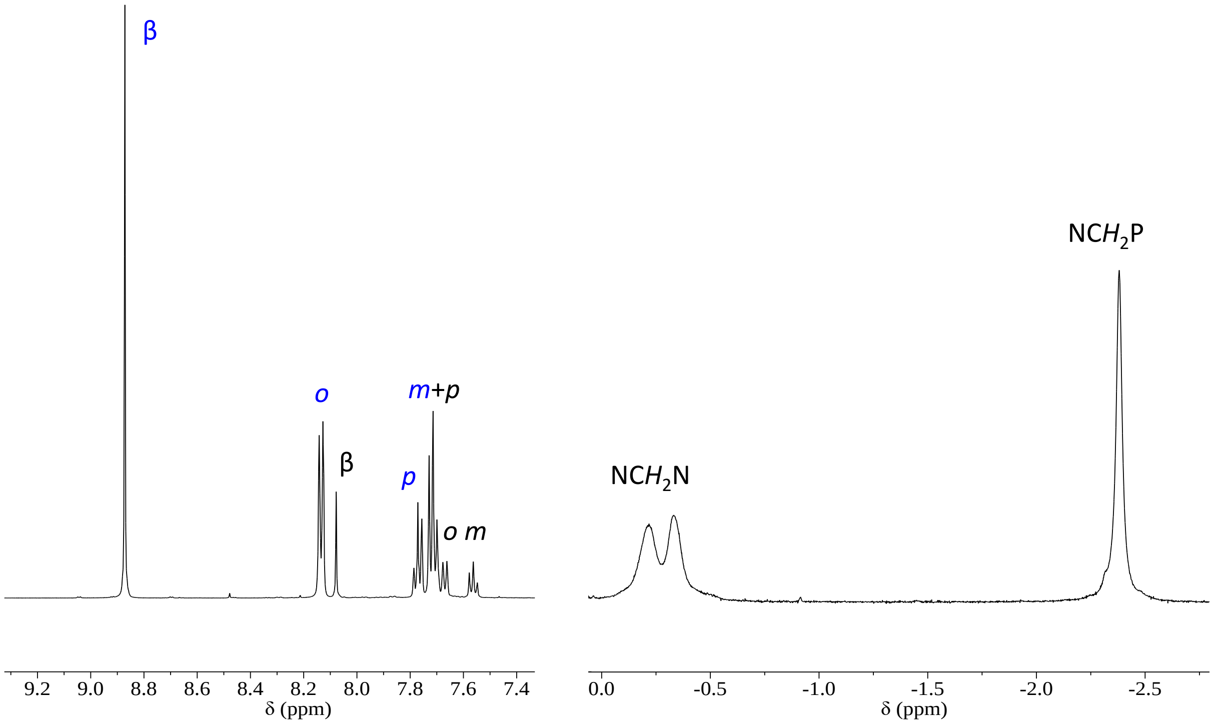

Figure S9. ^{1}H NMR spectrum (aromatic and PTA regions) of a 1:4:2 mixture of [Ru(TPP)(CO)] (black labels), Zn(TPP) (blue labels), and PTA. The downfield and upfield regions are not in scale: The PTA region has been amplified in order to better show the broad PTA resonances.

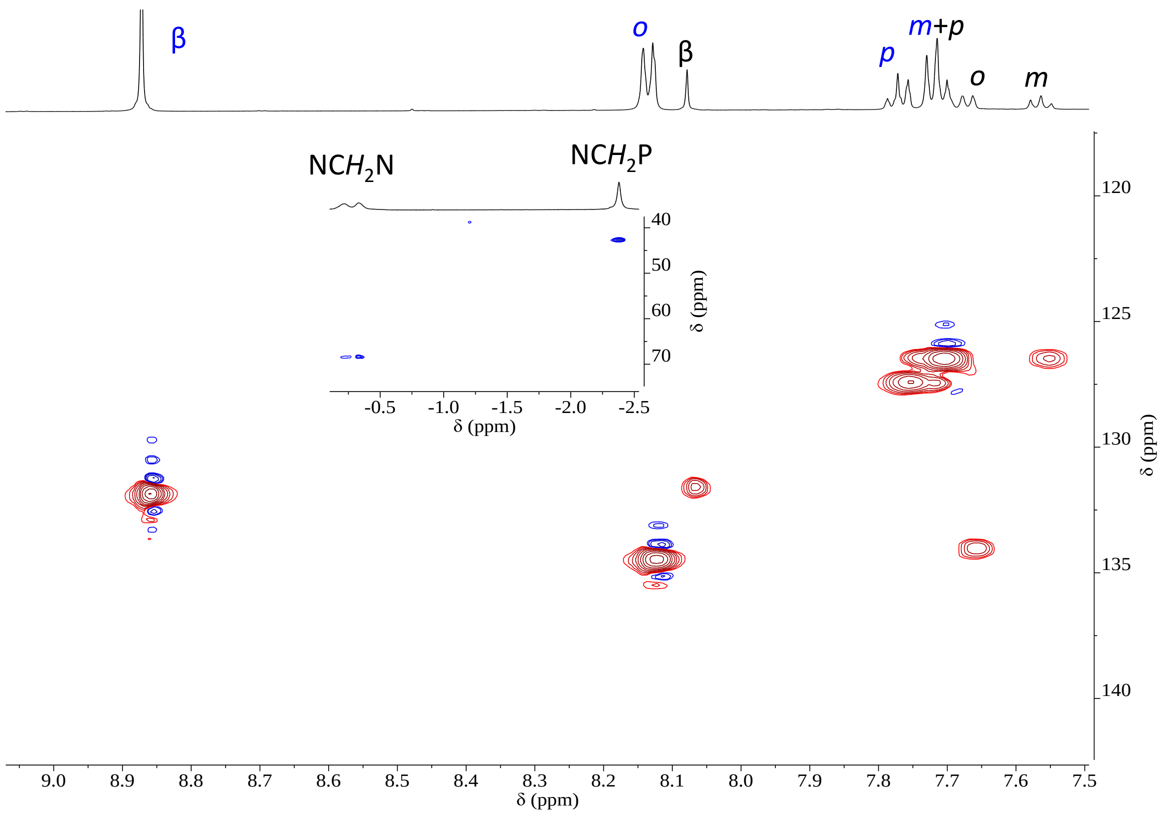

Figure S10. ^{1}H-^{13}C HSQC NMR spectrum of the 1:4:2 mixture of [Ru(TPP)(CO)] (black labels), Zn(TPP) (blue labels), and PTA. The PTA region is in the insert.

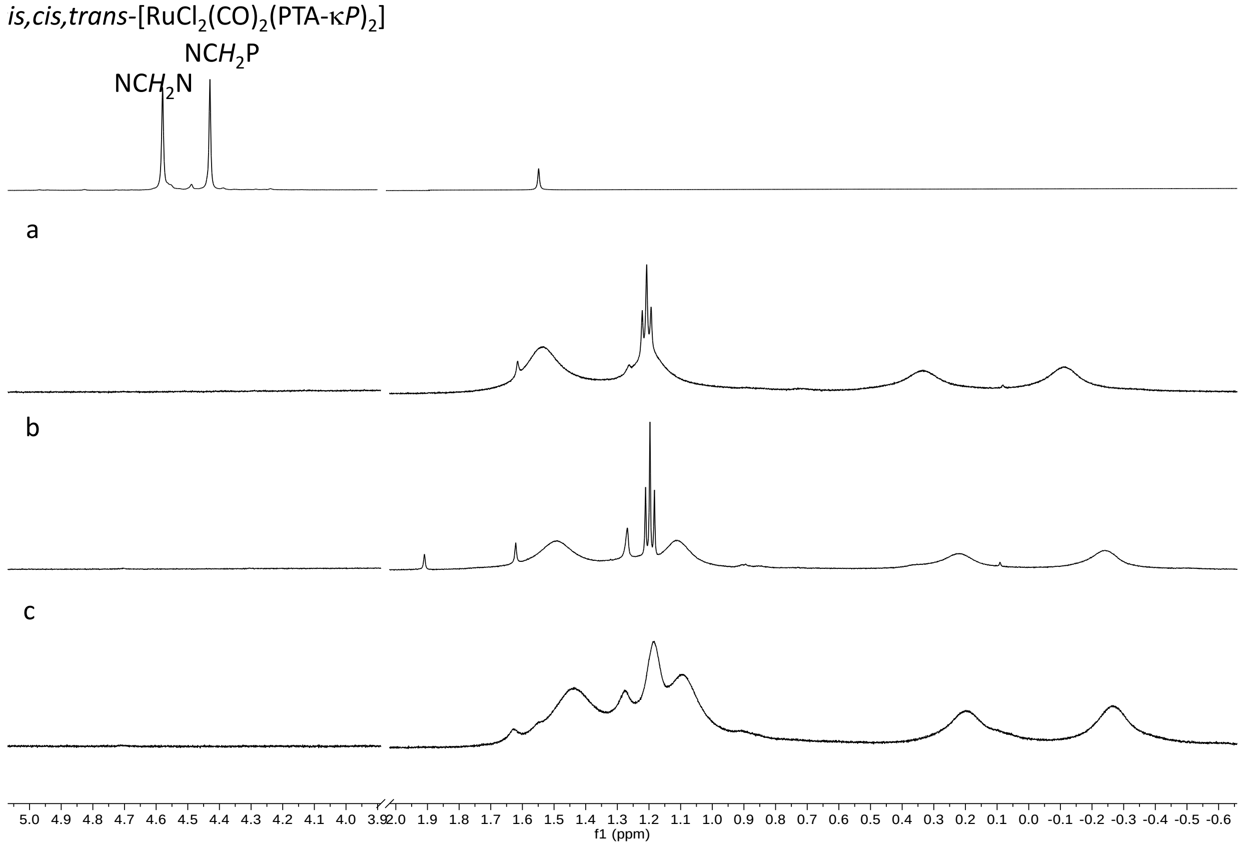

Figure S11. ^{1}H NMR titration of *cis,cis,trans*-[$RuCl_2(CO)_2$(PTA-κ*P*)$_2$] (**5**) (top) with 2 (a), 3 (b) and 4 (c) equiv of Zn(TPP) in $CDCl_3$. The porphyrin resonances are not shown. The triplet at ca. 1.2 ppm belongs to the EtOH originally bound to Zn(TPP).

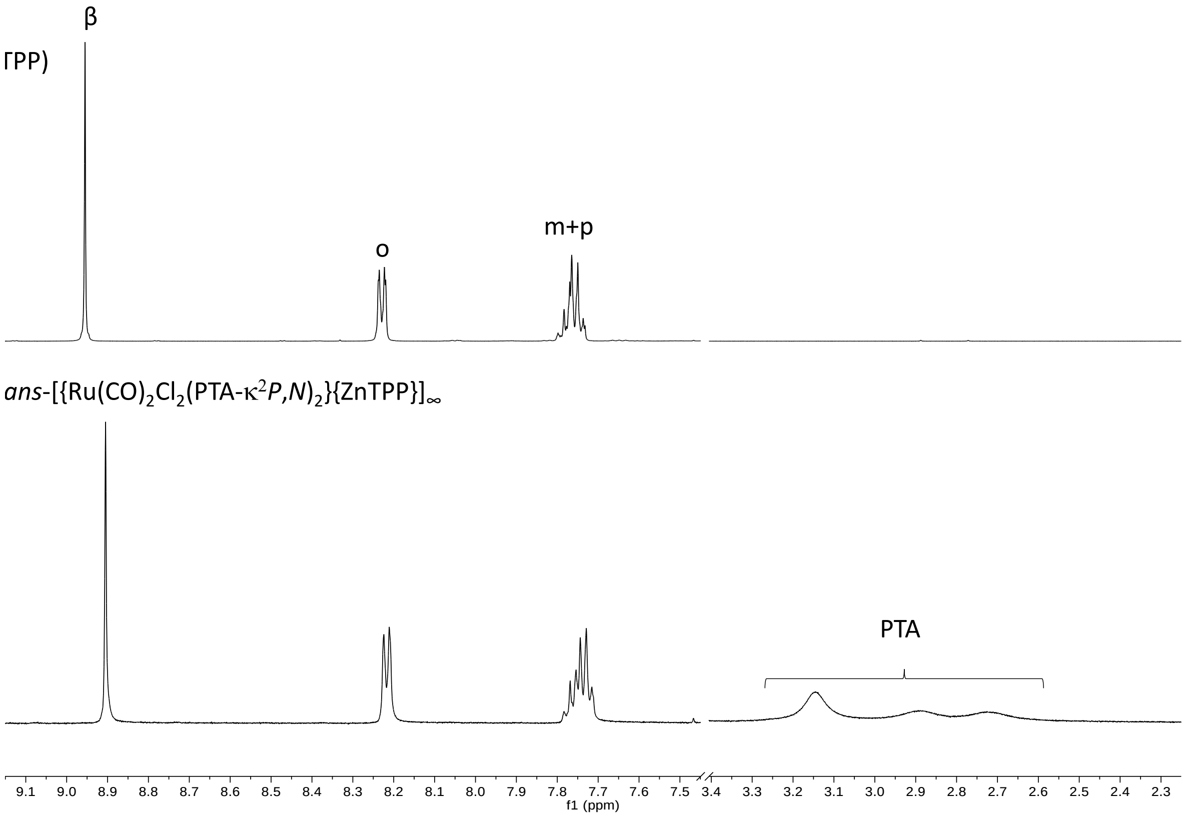

Figure S12. ^{1}H NMR spectrum of the crystals of *cis,cis,trans*-[{$RuCl_2(CO)_2$(PTA-κ^2*P*,*N*)$_2$}{ZnTPP}]$_\infty$ (**6**) dissolved in $CDCl_3$.

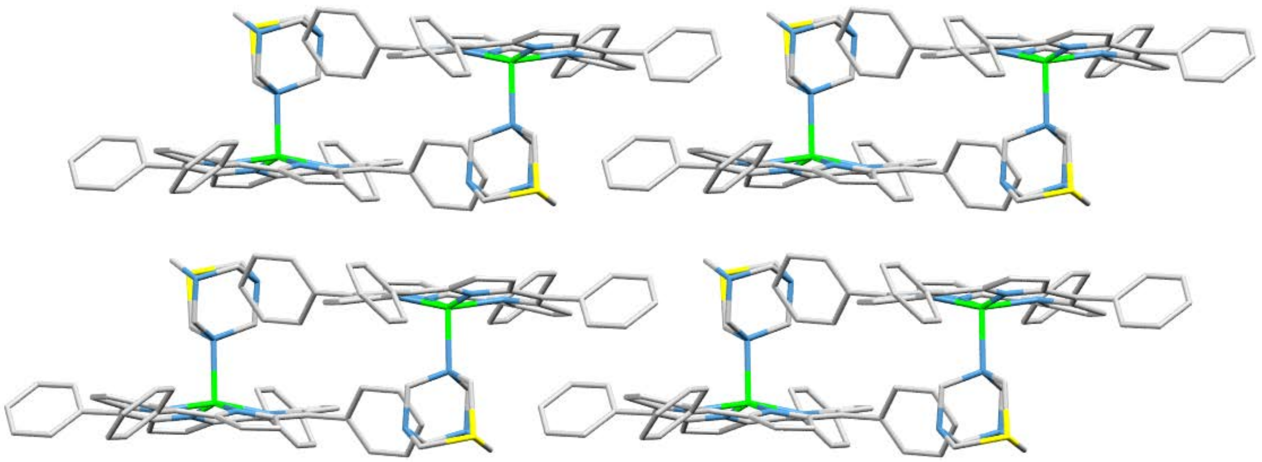

Figure S13. View along the b axis of a portion of the crystal structure of complex [Zn(TPP)(PTA-κ*N*)]·H_2O·$CHCl_3$ (**3**·H_2O·$CHCl_3$) evidencing the packing of the molecules and the κ*N* coordination of the PTA ligand to the Zn metal. Color code: C = grey, N = purple, P = orange. Crystallization molecules omitted.

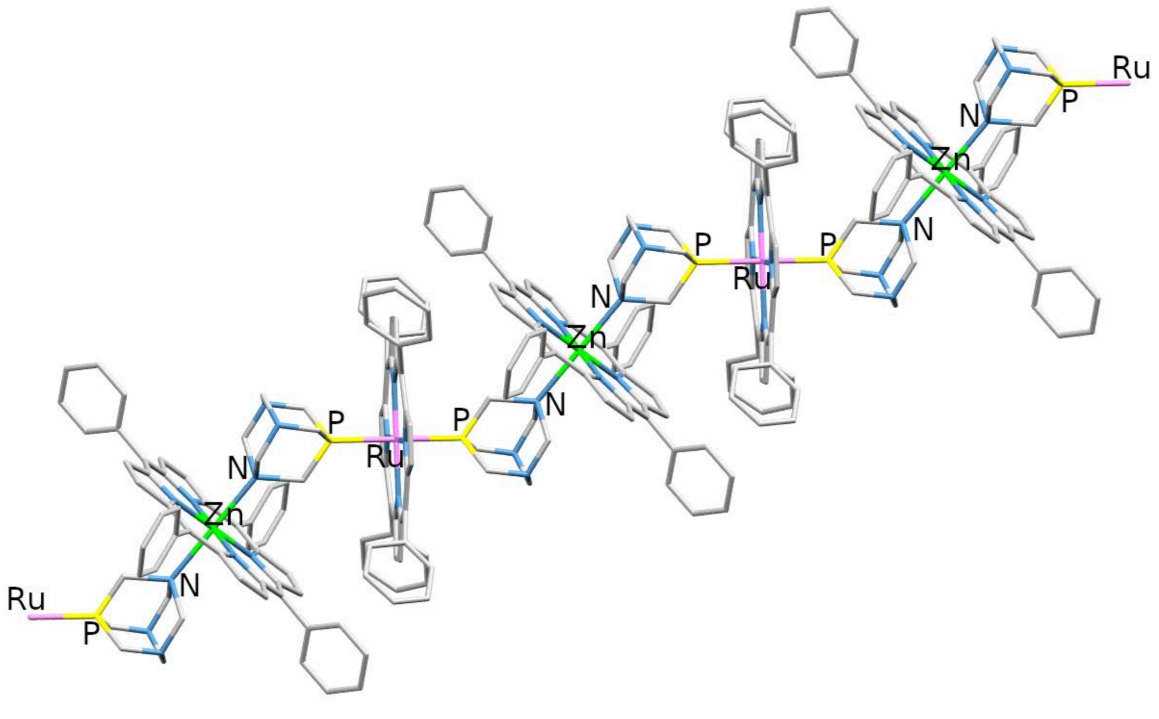

Figure S14. Stick representation of a portion of the zig-zag Ru(TPP)/Zn(TPP) chain present in the crystal structure of compound [{Ru(TPP)(PTA-κ^2*P*,*N*)$_2$}{Zn(TPP)}]$_\infty$ (**4**). Color code: Ru = light purple, Zn = green, P = yellow, N = blue.

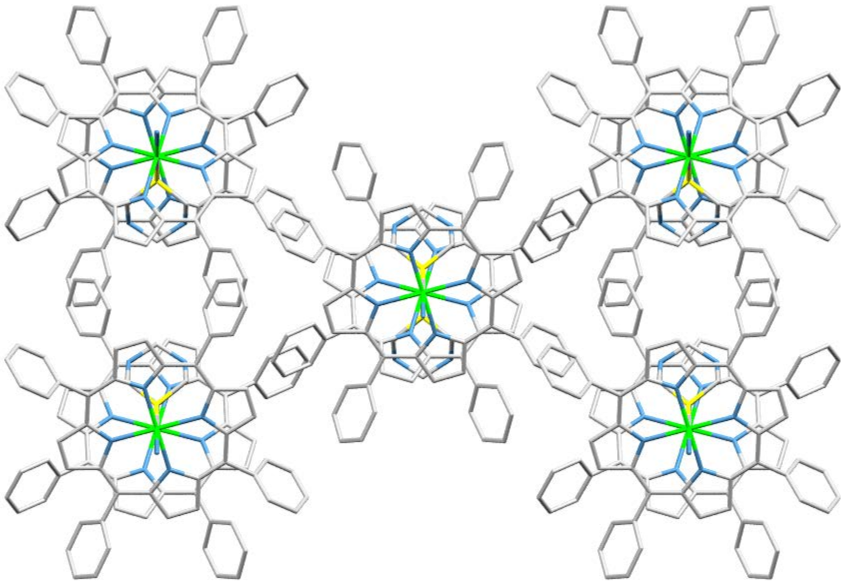

Figure S15. View along the c cell axis of five Ru(TPP)/Zn(TPP) chains in the crystal structure of compound [{Ru(TPP)(PTA-κ^2*P*,*N*)$_2$}{Zn(TPP)}]$_\infty$ (**4**).

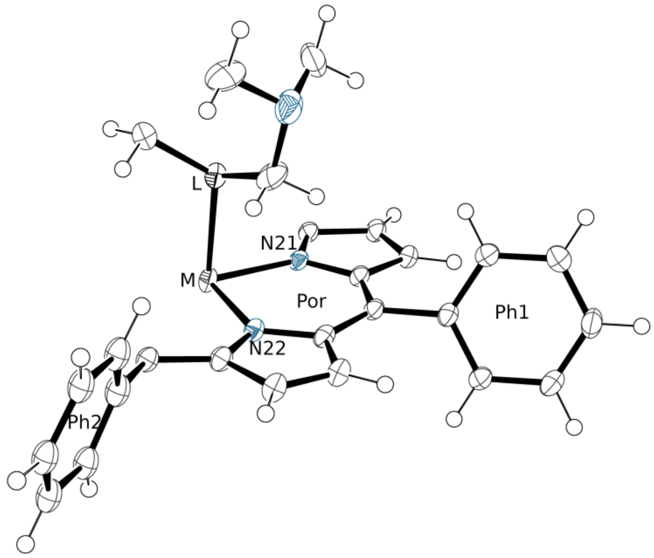

Figure S16. ORTEP representation (at 50% probability) of the asymmetric unit of the crystal structure of compound [{Ru(TPP)(PTA-κ^2*P*,*N*)$_2$}{Zn(TPP)}]$_\infty$ (**4**) (hydrogen atoms and minor population of a disordered phenyl group omitted for clarity). M and L indicate the crystallographic sites shared at 50% by Ru and Zn cations and P and N atoms, respectively; Por, Ph1 and Ph2 allow to visualize on the figure the dihedral angles reported in Table S4.

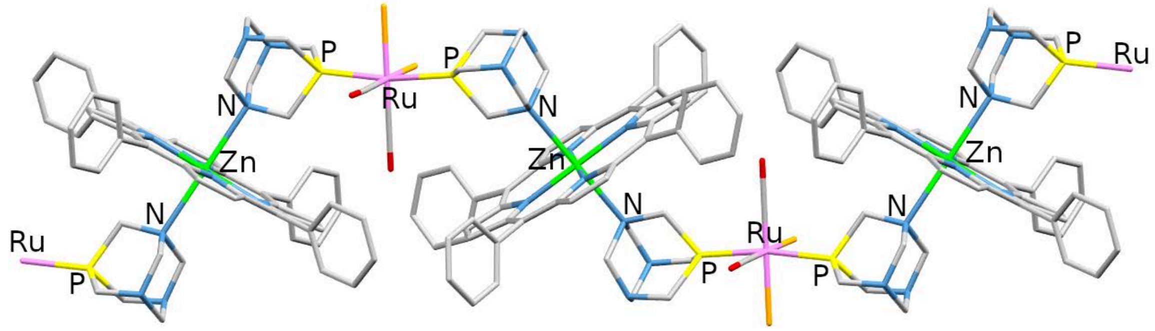

Figure S17. Stick representation of a portion of the linear "Greek frame" shaped Ru-Zn chain present in the crystal structure of compound *cis,cis,trans*-[{$RuCl_2(CO)_2$(PTA-$\kappa^2P,N)_2$}{Zn(TPP)}·9.2(H_2O)]$_\infty$ (**6**·9.2(H_2O)). Color code: Ru = light purple, Zn = green, P = yellow, N = blue, O = red, Cl = orange.

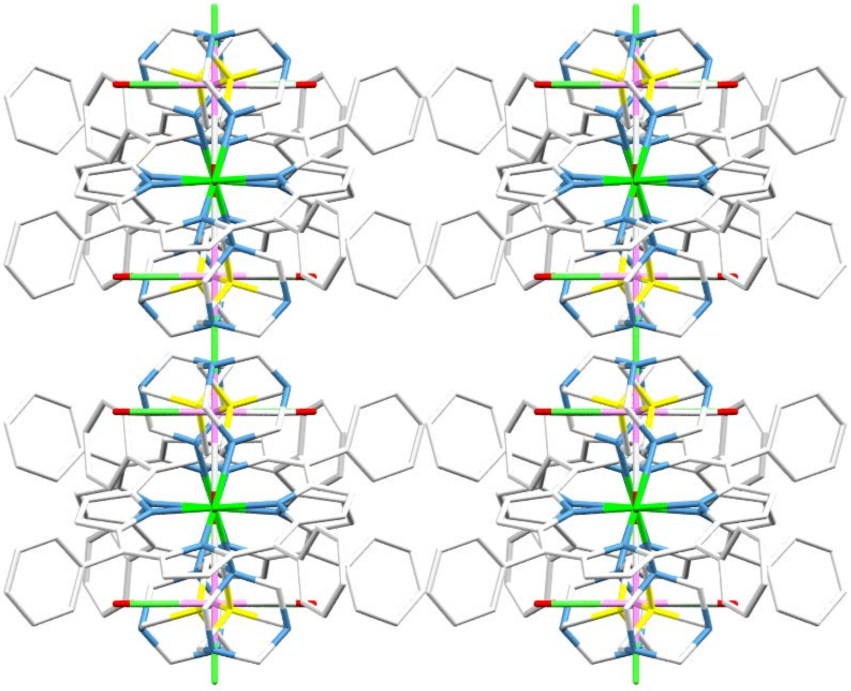

Figure S18. View along the c cell axis of four Ru-Zn chains in the crystal structure of compound *cis,cis,trans*-[{$RuCl_2(CO)_2$(PTA-$\kappa^2P,N)_2$}{Zn(TPP)}·9.2(H_2O)]$_\infty$ (**6**·9.2(H_2O)). The disordered water molecules – not shown – are located along the c axis, between the polymeric chains, and make hydrogen bonds among themselves but not with the chains. Color code: Ru = light purple, Zn = green, P = yellow, N = blue, O = red, Cl = orange.

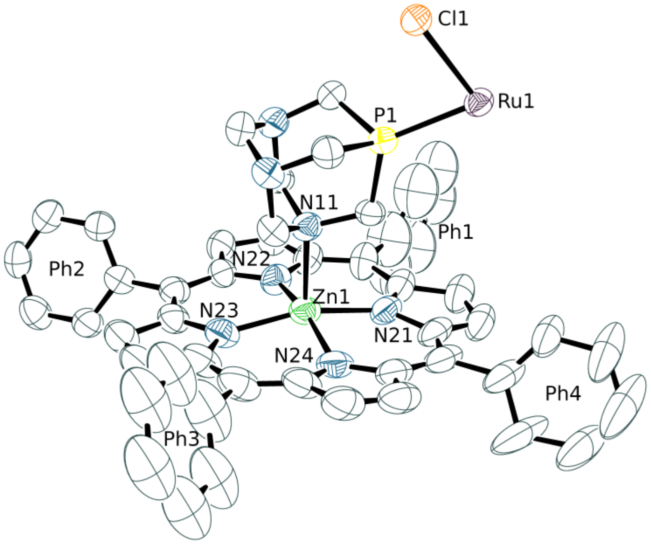

Figure S19. ORTEP representation (50% probability ellipsoids) of the asymmetric unit of the crystal structure of compound *trans*-[{$RuCl_2$(PTA-κ^2P,N)$_4$}{Zn(TPP)}$_4$]·8/3$CHCl_3$·2*n*-hexane (**8**·8/3$CHCl_3$·2*n*-hexane). Hydrogen atoms, two $CHCl_3$ and one *n*-hexane solvent molecules have been omitted for clarity. Labels PhX (X = 1 - 4) allow to visualize on the figure the dihedral angles quoted in Table S6. Color code: Ru = light purple, Zn = green, P = yellow, N = blue, O = red, Cl = orange.

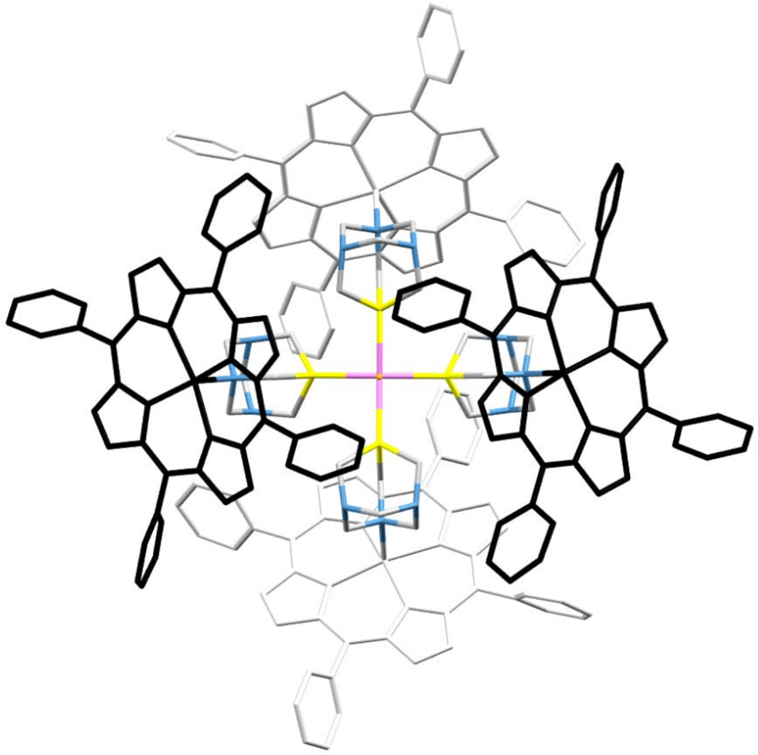

Figure S20. Stick representation of the molecule of complex *trans*-[{$RuCl_2$(PTA-κ^2P,N)$_4$}{Zn(TPP)}$_4$] (**8**) in the crystal structure viewed along the Cl–Ru–Cl axis. The ZnTPP units above and below the Ru equatorial plane have been evidenced with black and light gray colors, respectively.

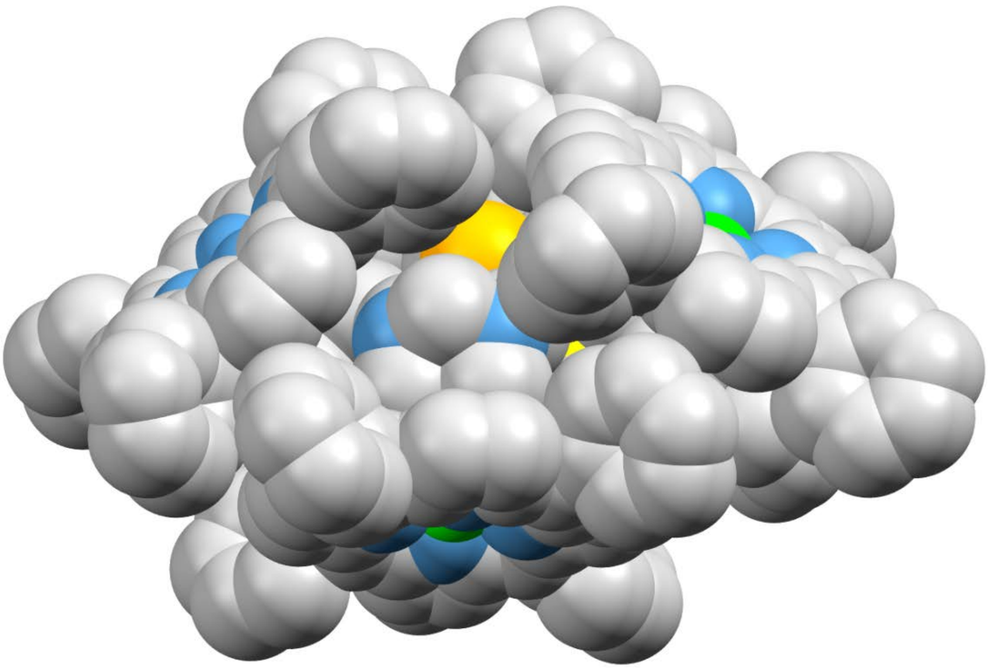

Figure S21. The space-fill representation of the molecule of complex *trans*-[{$RuCl_2$(PTA-κ^2*P*,*N*)$_4$}{Zn(TPP)}$_4$] (**8**) in the crystal structure evidences its very compact nature.

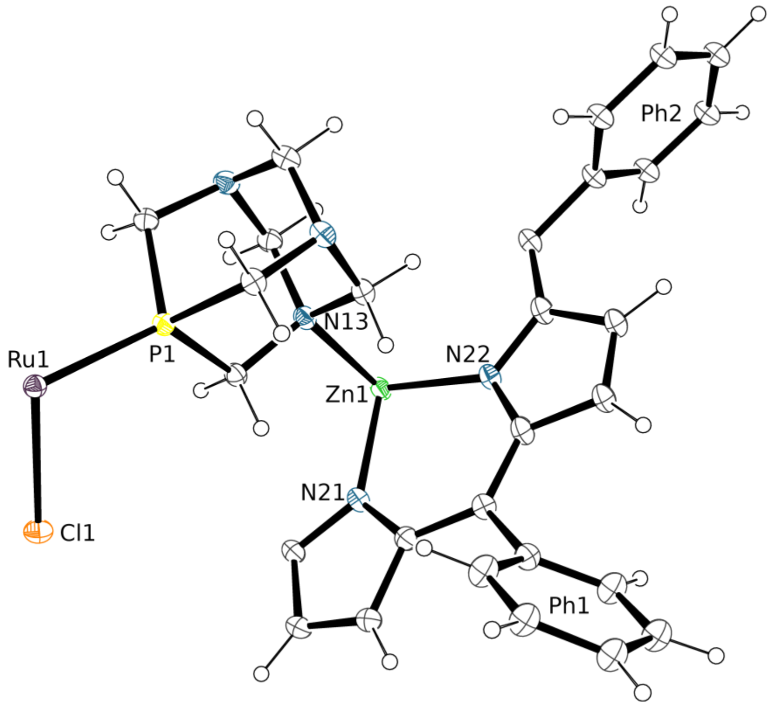

Figure S22. ORTEP representation (50% probability ellipsoids) of the asymmetric unit of the crystal structure of complex *trans*-[{$RuCl_2$(PTA-κ^2*P*,*N*)$_4$}{Zn(TPP)}$_2$·4$CHCl_3$]$_\infty$ (**9**·4$CHCl_3$). A disordered $CHCl_3$ solvent molecule has been omitted for clarity. For the same reason, only major populations of disordered phenyls and pyrrolic moieties of the TPP ligand have been included. Ph1 and Ph2 allow to visualize on the figure the dihedral angles reported in Table S7.

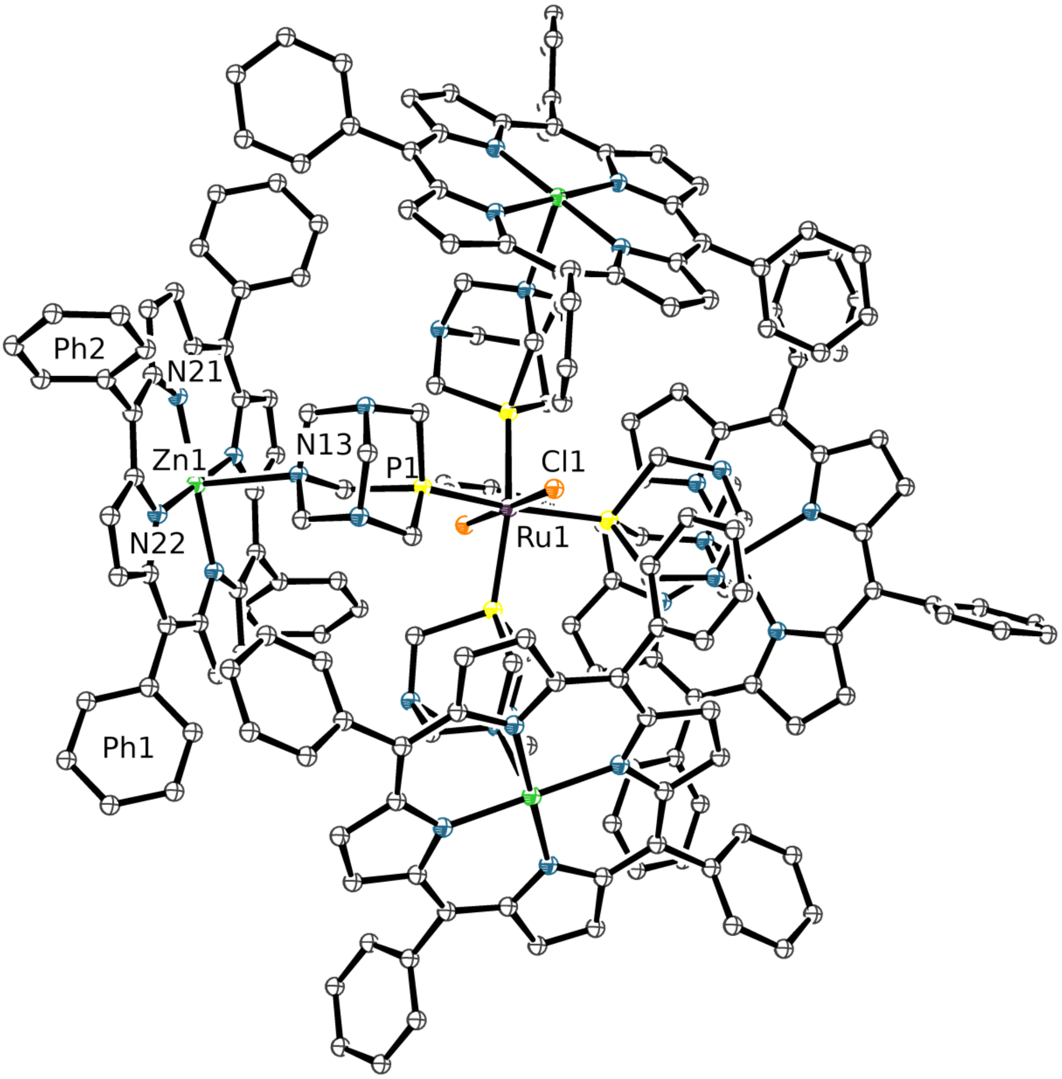

Figure S23. ORTEP representation (50% probability ellipsoids) of a "$RuZn_4$" fragment of the polymeric 3D net present in the crystal structure of complex *trans*-[{$RuCl_2$(PTA-κ^2*P*,*N*)$_4$}{Zn(TPP)}$_2$·4$CHCl_3$]$_\infty$ (**9**·4$CHCl_3$). Hydrogen atoms and a disordered $CHCl_3$ solvent molecule have been omitted for clarity. For the same reason, only major populations of disordered phenyls and pyrrolic moieties of the porphyrins have been included.

The zinc-acetate cluster of compound [{Ru(TPP)(PTA-κ^3*P,2N*)$_2$}{$Zn_9(CH_3COO)_{16}(CH_3OH)_2(OH)_2$}·9.2($H_2O$)]$_\infty$ (10·3$CHCl_3$).

The atomic arrangement of a single Zn_9 cluster of compound **10** is represented in Figure S24. There are nine Zn(II) cations, one of which, Zn4, occupies an inversion center. The remaining eight Zn cations are partitioned in two groups, related by the inversion center and therefore the description of the bonding environment is restrict to a single group of four Zn cations (Zn1, Zn2, Zn3, Zn5) plus Zn4 sitting on the inversion center. Overall, the Zn cations are connected to each other by bridging acetate anions, with some notable exceptions, as detailed below.

Zn4 has an octahedral environment in which an equatorial plane is formed by four O atoms (O311, O313, O311', O313') belonging to four distinct acetate anions, which provide bridging connections via their second O atom to Zn3, Zn5, Zn3' and Zn5', respectively. The axial positions of Zn4 are occupied by two (symmetry related) OH^- ions (O316 and O316'), that also cap in a μ_3 fashion Zn3 and Zn5 (O316), and Zn3' and Zn5' (O316').

Zn3 has a trigonal bipyramidal coordination with three O atoms (O35, O310, O314) from distinct bridging acetate anions in the equatorial plane, and axial positions occupied by the μ_3-hydroxo ligand mentioned above (O316) and an N atom (N22) of a PTA ligand *P*-bound to a Ru(TPP).

The coordination around Zn5 is less well defined. Zn5 is substantially five-coordinate by O atoms (equatorial: O37, O312, O316; apical: O39, O313), however the apical O313 is at a rather long distance of 2.473(4) Å (O313 also binds Zn4 at 2.126(4) Å). Moreover, a fourth weak interaction (bond length 2.570(4) Å) with an acetate O atom (O38) is also present in the equatorial plane.

Zn2 is connected via acetate bridges to both Zn3 and Zn5. Its octahedral coordination environment is provided by five O atoms of distinct bridging acetate ions (O31, O33, O36, O38, and O318) and a methanol ligand (O315). As mentioned above, O38 is also involved in an additional weak interaction with Zn5. Zn1 has a tetrahedral coordination, which is provided by the O atoms (O32, O33, and O317) of three distinct bridging acetate ions that connect Zn1 to Zn2, and by an N atom (N21) of a PTA ligand P-bound to a Ru(TPP).

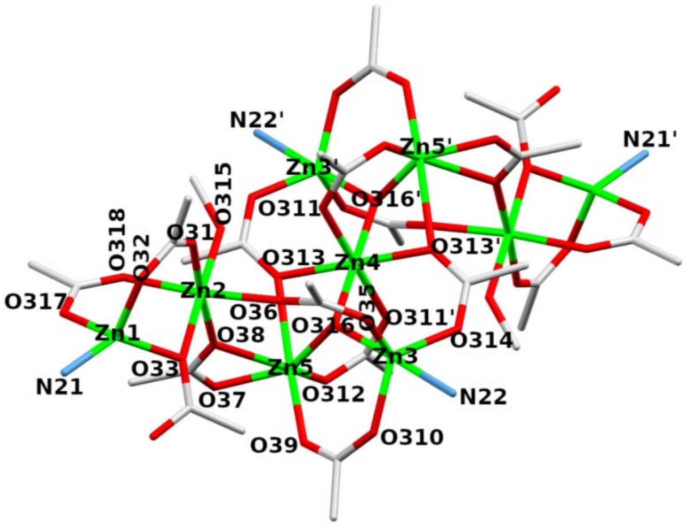

Figure S24. Stick representation of the Zn_9 cluster in the crystal structure of compound [{Ru(TPP)(PTA-κ^3*P*,*2N*)$_2$}{$Zn_9(CH_3COO)_{16}(CH_3OH)_2(OH)_2$}·$3CHCl_3$]$_\infty$ (**10**·$3CHCl_3$) (hydrogen atoms omitted for clarity). The nitrogen atoms of the four PTA ligands to which the Zn cluster binds are also shown. Primed atoms are symmetry images of corresponding non primed atoms via the inversion center at Zn4. Color code: Zn = green, N = blue, O = red.

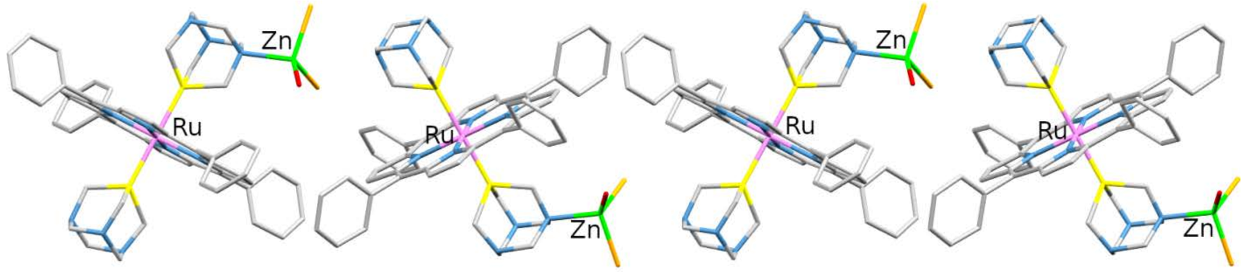

Figure S25. Stick representation of a portion of the 1D sequence present in the crystal structure of compound [{Ru(TPP)(PTA-κ*P*)(PTA-κ^2*P*,*N*)}{$ZnCl_2(OH_2)$}] (**11**). Color code: Ru = purple, Zn = green, P = yellow, N = blue, Cl = orange, O = red.

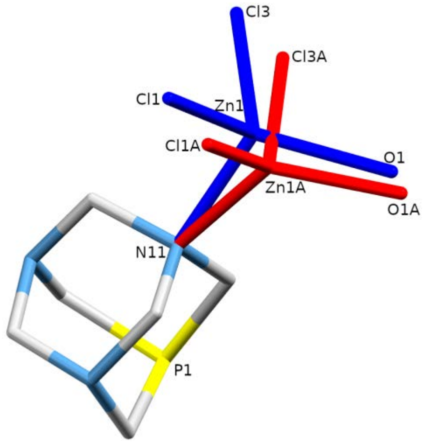

Figure S26. Stick representation of the disordered $ZnCl_2(H_2O)$ group in the crystal structure of compound [{Ru(TPP)(PTA-κ*P*)(PTA-κ^2*P*,*N*)}{$ZnCl_2(OH_2)$}] (**11**). The two populations are evidenced in blue (SOF=0.3) and red (SOF=0.2). The symmetry mates generated by a 2-fold axis close to the Zn atoms (see text) have been omitted for clarity.

Additional comments on the X-ray structure of [{Ru(TPP)(PTA-κ*P*)(PTA-κ^2*P*,*N*)}{$ZnCl_2(OH_2)$}] (11)

As said in the main text, due to the low quality of the X-ray data and to the large disorder found around the Zn atom (Figure S26), the observed Fourier map for compound **11** could be refined also with a model consisting of the zwitterionic molecule [{Ru(TPP)(PTAH-κ*P*)(PTA-κ^2*P*,*N*)}$^+${$ZnCl_3$}$^-$]. We believe that chloroform, which is known to generate small amounts of HCl in the presence of oxygen and light,[S1] could be a plausible source of the adventitious protons for the generation of $PTAH^+$. In this hypothesis, adjacent zwitterions would be connected by the electrostatic interaction of the $ZnCl_3^-$ group of one with the $PTAH^+$ ligand of the other, forming a 1D ionic polymer. No similar structures can be found in the literature. However, crystallization of hexamethylenetetramine (HTMA) with $ZnCl_2$ in aqueous ammonia afforded $(NH_4)[ZnCl_3(HTMA)]\cdot 1.5H_2O$,[S2] whereas crystals of $[Zn(OH_2)_6][ZnCl_3(HTMA)]_2\cdot 0.5H_2O$ were obtained from an aqueous solution containing equimolar amounts of Zn^{2+}, Cl^- and HTMA.[S3]

Even though the X-ray data are not conclusive about the nature of compound **11**, we prefer the “neutral” formulation [{Ru(TPP)(PTA-κ*P*)(PTA-κ^2*P*,*N*)}{$ZnCl_2(OH_2)$}] over the zwitterionic one for the following reasons: 1) it is more chemically reasonable, since it does not require protonation of PTA in the absence of added acid; 2) the *R* factor of the X-ray structure is slightly better (8.4 vs 9.4); 3) the Zn–ligand bond lengths are more consistent with literature data. In fact, in the $ZnCl_3^-$ hypothesis, one of the three Zn–Cl bond lengths is particularly short (2.199(7) Å vs 2.239(4) and 2.25(1) Å for the other two)

Finally, we note that as far as the binding behavior of PTA is concerned, the two structural solutions are very similar.

References

[S1] a) Hill, B. G. Photochemical decomposition of chloroform *J. Am. Chem. Soc.* **1932**, *54*, 32-40; b) Kawai, S. Discussion on decomposition of chloroform *J-Stage* **1966**, *86*, 1125-1132.

[S2] Cheng, Y.-Q.; Lv, L.-P.; Xie, J.-W.; Wang, H.-B.; Jin, Z.-M. Ammonium trichloro(hexamethylenetetramine) zincate(II) sesquihydrate *Acta Crystallogr., Sect. E: Struct. Rep. Online* **2006**, *62*, m3591-m3593.

[S3] Basdouri, Z.; Trojette, B.; Falvello, L. R.; Graia, M.; Tomas, M. Synthesis, crystal structure, infrared spectroscopy, thermal analysis and Hirshfeld surface analysis of a new hemihydrate of $[Zn(H_2O)_6][\{(CH_2)_6N_4\}ZnCl_3]_2\cdot 0.5H_2O$ *J. Mol. Struct.* **2019**, *1176*, 165-180.

Table S1. Crystallographic data and refinement details for compounds [Ru(TPP)(PTA-κ*P*)$_2$]·2CHCl$_3$ (**1**·2CHCl$_3$) and [Zn(TPP)(PTA-κ*N*)]·H$_2$O·CHCl$_3$ (**3**·H$_2$O·CHCl$_3$).

	**1**·2CHCl$_3$	**3**·H$_2$O·CHCl$_3$
Empirical Formula	$C_{56}H_{52}N_{10}P_2Ru\cdot 2CHCl_3$	$C_{50}H_{40}N_7PZn\cdot H_2O\cdot CHCl_3$
Formula weight (Da)	1266.82	1945.31
Temperature (K)	173(2)	100(2)
Wavelength (Å)	0.700	0.700
Crystal system	monoclinic	triclinic
Space Group	*P* 21/c	*P* –1
a (Å)	13.770(5)	8.752(2)
b (Å)	18.932(4)	14.364(3)
c (Å)	10.952(2)	19.295(4)
α (°)	90	109.39(3)
β (°)	98.71(2)	100.96(3)
γ (°)	90	97.50(3)
V (Å^3)	2822(1)	2196.6(9)
Z	2	2
ρ (g·cm^{-3})	1.491	1.470
F(000)	1296	1004
μ (mm^{-1})	0.632	0.790
θ min, max (°)	1.815, 29.084	1.514, 28.227
Resolution (Å)	0.72	0.74
Total refl. collctd	49719	20757
Independent refl.	7811	10915
Obs. Refl. [Fo>4σ(Fo)]	7522	10630
I/σ(I) (all data)	18.75	52.26
I/σ(I) (max res)	15.92	36.70
Completeness (all data)	0.981	0.962
R_{merge} (all data)	7.3%	3.1%
R_{merge} (max res)	7.8%	3.3%
Multiplicity (all data)	6.3	19.2
Multiplicity (max res)	6.0	6.2
Data/restraint/parameters	7811/0/350	10915/98/678
GooF	1.050	1.063
R[I>2.0σ(I)],[a] wR2 [I>2.0σ(I)][a]	0.0453, 0.1255	0.0511, 0.1309
R (all data),[a] wR2 (all data)[a]	0.0460, 0.1263	0.0522, 0.1319

[a] $R_1 = \Sigma\, ||Fo|-|Fc|| / \Sigma|Fo|$, $wR_2 = [\Sigma\, w\, (Fo^2 - Fc^2)^2 / \Sigma w\, (Fo^2)^2]^{½}$

Table S1cont. Crystallographic data and refinement details for compounds [{Ru(TPP)(PTA-κ^2*P*,*N*)$_2$}{Zn(TPP)}]$_\infty$ (**4**) and *cis,cis,trans*-[{$RuCl_2(CO)_2$(PTA-κ*P*,*N*)$_2$}{Zn(TPP)}·9.2(H_2O)]$_\infty$ (**6**·9.2(H_2O)).

	**4**	**6**·9.2(H_2O)
Empirical Formula	$C_{100}H_{80}N_{14}P_2RuZn$	$C_{58}H_{52}N_{10}Cl_2O_2P_2RuZn\cdot$ 9.2(H_2O)
Formula weight (Da)	1706.16	2606.49
Temperature (K)	100(2)	100(2)
Wavelength (Å)	0.700	0.700
Crystal system	monoclinic	monoclinic
Space Group	C 2/c	*P* 2/c
a (Å)	23.524(3)	13.067(1)
b (Å)	11.925(4)	10.149(3)
c (Å)	14.409(3)	24.8580(9)
α (°)	90	90
β (°)	98.200(4)	91.568(5)
γ (°)	90	90
V ($Å^3$)	4001(2)	3295(1)
Z	4	1
ρ ($g\cdot cm^{-3}$)	1.416	1.313
F(000)	1764	1340
μ (mm^{-1})	0.563	0.740
θ min, max (°)	2.281, 28.225	2.552, 29.084
Resolution (Å)	0.74	0.72
Total refl. collctd	62648	57176
Independent refl.	5127	9173
Obs. Refl. [Fo>4σ(Fo)]	4650	8477
I/σ(I) (all data)	31.39	16.62
I/σ(I) (max res)	20.55	11.92
Completeness (all data)	0.986	0.991
R_{merge} (all data)	6.3%	7.4%
R_{merge} (max res)	17.2%	10.7%
Multiplicity (all data)	12.0	6.1
Multiplicity (max res)	11.4	5.8
Data/restraint/parameters	5127/0/256	9173/23/343
GooF	1.062	1.046
R[I>2.0σ(I)],[a] wR2 [I>2.0σ(I)][a]	0.0341,0.0895	0.0684, 0.1910
R (all data),[a] wR2 (all data)[a]	0.0374,0.0914	0.0715,0.1950

$^aR_1 = \Sigma\, |Fo|–|Fc|| / \Sigma|Fo|$, $wR_2 = [\Sigma\, w\, (Fo^2 – Fc^2\,)^2 / \Sigma w\, (Fo^2\,)^2]^{½}$

Table S1cont. Crystallographic data and refinement details for compounds *trans*-[$RuCl_2$(PTA-κ^2*P*,*N*)$_4${Zn(TPP)}$_4$]·8/3$CHCl_3$·2*n*-hexane (**8**·8/3$CHCl_3$·2*n*-hexane) and *trans*-[{$RuCl_2$(PTA-κ^2*P*,*N*)$_4$}{Zn(TPP)}$_2$·4$CHCl_3$]$_\infty$ (**9**·4$CHCl_3$).

	**8**·8/3$CHCl_3$·2*n*-hexane	**9**·4$CHCl_3$
Empirical Formula	$C_{200}H_{160}N_{28}Cl_2P_4RuZn_4$·8/3$CHCl_3$·2$C_6H_{14}$	$RuZn_2Cl_2P_4C_{112}H_{104}N_{20}$·4$CHCl_3$
Formula weight (Da)	4003.54	2634.21
Temperature (K)	100(2)	100(2)
Wavelength (Å)	0.700	0.700
Crystal system	cubic	tetragonal
Space Group	I –4 3 d	I 41/a
a (Å)	38.537(7)	27.014(5)
b (Å)	38.537(7)	27.014(5)
c (Å)	38.537(7)	15.507(2)
α (°)	90	90
β (°)	90	90
γ (°)	90	90
V ($Å^3$)	57231(29)	11316(4)
Z	12	4
ρ ($g \cdot cm^{-3}$)	1.394	1.546
F(000)	24824	5384
μ (mm^{-1})	0.773	0.951
θ min, max (°)	1.275, 26.656	1.485, 29.080
Resolution (Å)	0.78	0.72
Total refl. collctd	389280	101816
Independent refl.	10529	7932
Obs. Refl. [Fo>4σ(Fo)]	10195	7695
I/σ(I) (all data)	77.13	67.79
I/σ(I) (max res)	23.75	45.42
Completeness (all data)	1.000	1.000
R_{merge} (all data)	4.7%	2.7%
R_{merge} (max res)	27.2%	3.7%
Multiplicity (all data)	68.7	12.8
Multiplicity (max res)	69.4	12.3
Data/restraint/parameters	10529/49/498	7932/19/348
GooF	1.031	1.033
R[I>2.0σ(I)],[a] wR2 [I>2.0σ(I)][a]	0.0785, 0.2284	0.0478, 0.1390
R (all data),[a] wR2 (all data)[a]	0.0798, 0.2317	0.0485,0.1397

[a] $R_1 = \Sigma \,||Fo|-|Fc|| / \Sigma|Fo|$, $wR_2 = [\Sigma\, w\, (Fo^2 - Fc^2)^2 / \Sigma w\, (Fo^2)^2]^{½}$

Table S1cont. Crystallographic data and refinement details for compounds [{Ru(TPP)(PTA-κ^3*P*,*2N*)$_2$}{$Zn_9(CH_3COO)_{16}(CH_3OH)_2(OH)_2$}·$3CHCl_3$]$_\infty$ (**10**·$3CHCl_3$) and [{Ru(TPP)(PTA-κ*P*)(PTA-κ^2*P*,*N*)}{$ZnCl_2(OH_2)$}·$0.6CHCl_3$] (**11**·$0.6CHCl_3$).

	**10**·$3CHCl_3$	**11**·$0.6CHCl_3$
Empirical Formula	$C_{56}H_{52}N_{10}P_2Ru$ + $C_{34}H_{58}O_{36}Zn_9$·$3CHCl_3$	$C_{56}H_{54}N_{10}Cl_2OP_2RuZn$·$0.6CHCl_3$
Formula weight (Da)	1508.66	1253.99
Temperature (K)	100(2)	100(2)
Wavelength (Å)	0.700	0.700
Crystal system	triclinic	monoclinic
Space Group	*P* –1	C 2/c
a (Å)	14.04(1)	17.404(4)
b (Å)	15.457(5)	19.878(5)
c (Å)	17.954(6)	17.524(2)
α (°)	110.15(1)	90
β (°)	109.19(2)	91.143(8)
γ (°)	94.31(2)	90
V (Å^3)	3375(4)	6061(2)
Z	1	4
ρ (g·cm^{-3})	1.484	1.347
F(000)	1526	2563
μ (mm^{-1})	1.866	0.868
θ min, max (°)	1.414, 28.227	1.899, 28.227
Resolution (Å)	0.74	0.74
Total refl. collctd	111508	89921
Independent refl.	17166	7375
Obs. Refl. [Fo>4σ(Fo)]	10951	6547
I/σ(I) (all data)	8.96	19.02
I/σ(I) (max res)	1.79	8.40
Completeness (all data)	0.982	0.922
R_{merge} (all data)	13.5%	8.5%
R_{merge} (max res)	136.0%	24.7%
Multiplicity (all data)	6.5	11.9
Multiplicity (max res)	6.2	11.8
Data/restraint/parameters	17166/0/746	7375/96/469
GooF	1.028	1.002
R[I>2.0σ(I)],[a] wR2 [I>2.0σ(I)][a]	0.0658, 0.1697	0.0840, 0.2396
R (all data),[a] wR2 (all data)[a]	0.1075, 0.1952	0.0893, 0.2481

[a] $R_1 = \Sigma \left| |Fo| - |Fc| \right| / \Sigma |Fo|$, $wR_2 = [\Sigma\, w\, (Fo^2 - Fc^2)^2 / \Sigma w\, (Fo^2)^2]^{½}$

Table S2. Selected coordination distances (Å) and angles (°) for [Ru(TPP)(PTA-κ*P*)$_2$]·2$CHCl_3$ (**1**·2$CHCl_3$).

**Bond distances (Å)**			
Ru1–N1	2.064(1)	Ru1–P1	2.3253(7)
Ru1–N2	2.056(2)		
**Bond angles (°)**			
N1–Ru1–P1	88.04(4)	N2–Ru1–P1	88.82(5)
N1–Ru1–N2	90.09(6)		
**Dihedral angles (°)**			
Ru(TPP)··· Ph1	68.60(4)	Ru(TPP)··· Ph2	63.45(5)

The Ru atom sits on an inversion center, therefore some distances/angles are fixed (e.g. angle N1–Ru–N1' = 180°) and are not reported in the Table.

Table S3. Selected coordination distances (Å) and angles (°) for [Zn(TPP)(PTA-κ*N*)]·H_2O·$CHCl_3$ (**3**·H_2O·$CHCl_3$).

**Bond distances (Å)**			
Zn1–N1	2.079(2)	Zn1–N3	2.068(2)
Zn1–N2	2.070(2)	Zn1–N4	2.067(2)
Zn1–N21	2.186(2)		
**Bond angles (°)**			
N1–Zn1–N21	97.74(7)	N3–Zn1–N21	100.14(7)
N2–Zn1–N1	88.13(6)	N4–Zn1–N1	88.16(6)
N2–Zn1–N21	99.11(7)	N4–Zn1–N2	159.47(6)
N3–Zn1–N1	162.10(6)	N4–Zn1–N3	89.10(6)
N3–Zn1–N2	88.25(6)	N4–Zn1–N21	101.40(7)
**Dihedral angles (°)**			
Zn(TPP)··· Ph1	46.41(4)	Zn(TPP)··· Ph2	50.95(4)
Zn(TPP)··· Ph3	62.28(5)	Zn(TPP)··· Ph4	43.19(4)

Table S3bis. Selected bond distances (Å) for the PTA ligand in [Zn(TPP)(PTA-κ*N*)]·H_2O·$CHCl_3$ (**3**·H_2O·$CHCl_3$).

**Bond distances (Å)**			
N21–C21	1.487(3)	N23–C23	1.463(4)
N21 C24	1.496(3)	N23–C25	1.447(4)
N21 C26	1.493(3)	N23–C26	1.451(3)
N22–C22	1.468(3)	P21–C21	1.860(2)
N22–C24	1.458(3)	P21–C22	1.854(3)
N22–C25	1.471(3)	P21–C23	1.855(3)

Table S4. Selected coordination distances (Å) and angles (°) for [{Ru(TPP)(PTA-κ^2*P*,*N*)$_2$}{Zn(TPP)}]$_\infty$ (**4**).

**Bond distances (Å)**			
M–N21	2.049(1)	M–L	2.3800(7)
M–N22	2.062(1)		
**Bond angles (°)**			
N21–M–N22	89.92(5)	N22–M–L	89.00(4)
N21–M–L	88.46(4)		
**Dihedral angles (°)**			
M(TPP)··· Ph1	62.97(5)	M(TPP)··· Ph2	76.80(8)

Since in **4** the equatorial environment of Ru and Zn is identical and the P/N bonding modes of the PTA ligand are nearly geometrically equivalent, the symmetry of the observed diffraction pattern (space group C2/c) does not distinguish the two metal ions and the corresponding PTA binding modes leading to a crystallographically independent fragment in which a single metal site (M) is equally partitioned between Ru and Zn and, correspondingly, two symmetry related binding sites (L) of the PTA are partitioned at 50% between P and N. See also Figure S14 and S16.

Table S5. Selected coordination distances (Å) and angles (°) for *cis*,*cis*,*trans*-[{$RuCl_2(CO)_2$(PTA-$\kappa^2 P,N)_2$}{Zn(TPP)}·9.2(H_2O)]$_\infty$ (**6**·9.2(H_2O)).

**Bond distances (Å)**			
Ru1–C1	1.907(5)	Ru1–P1	2.3480(7)
Ru1–C2	1.960(1)	Zn1–N1	2.059(2)
Ru1–Cl1	2.414(1)	Zn1–N2	2.058(2)
Ru1–Cl2	2.285(2)	Zn1–N11	2.532(2)
**Bond angles (°)**			
C1–Ru1–C2	89.6(3)	Cl2–Ru1–P1	87.74(7)
C1–Ru1–Cl2	90.29(7)	P1–Ru1–Cl1	85.41(2)
C1–Ru1–P1	94.59(2)	N1–Zn–N2	90.70(9)
C2–Ru1–Cl1	90.4(3)	N1–Zn–N2′	89.30(9)
C2–Ru1–P1	89.9(4)	N1–Zn–N11	87.77(8)
Cl2–Ru1–Cl1	89.71(7)	N2–Zn–N11	89.53(9)
**Dihedral angles (°)**			
Zn(TPP)··· Ph1	77.5(1)	Zn(TPP)··· Ph2	65.1(1)

Table S6. Selected coordination distances (Å) and angles (°) for *trans*-[RuCl$_2$(PTA-κ^2*P*,*N*)$_4${Zn(TPP)}$_4$]·8/3CHCl$_3$·2*n*-hexane (**8**·8/3CHCl$_3$·2*n*-hexane).

**Bond distances (Å)**			
Ru1–P1	2.324(2)	Zn1–N22	2.066(6)
Ru1–Cl1	2.412(2)	Zn1–N21	2.069(8)
Zn1–N11	2.242(6)	Zn1–N24	2.078(7)
Zn1–N23	2.044(8)		
**Bond angles (°)**			
P1–Ru1–Cl1	80.10(4)		
**Dihedral angles (°)**			
Zn(TPP)··· Ph1	78.1(2)	Zn(TPP)··· Ph3	63.2(3)
Zn(TPP)··· Ph2	68.2(2)	Zn(TPP)··· Ph4	55.5(4)

Table S7. Selected coordination distances (Å) and angles (°) for *trans*-[{$RuCl_2$(PTA-κ^2*P*,*N*)$_4$}{Zn(TPP)}$_2$·$4CHCl_3$]$_\infty$ (**9**·$4CHCl_3$).

**Bond distances (Å)**			
Ru1–P1	2.3359(7)	Zn1–N21	2.063(2)
Ru1–Cl1	2.4246(8)	Zn1–N22	2.055(2)
Zn1–N13	2.4869(2)		
**Bond angles (°)**			
P1–Ru1–Cl1	100.60(1)	N22–Zn1–N13	89.78(7)
N21–Zn1–N13	87.83(7)	N21–Zn1–N22	89.74(7)
**Dihedral angles (°)**			
Zn(TPP)··· Ph1	60.20(8)	Zn(TPP)··· Ph2	73.90(8)

Table S8. Selected coordination distances (Å) and angles (°) for the {Ru(TPP)} part of [{Ru(TPP)(PTA-κ^3*P*,*2N*)$_2$}{$Zn_9(CH_3COO)_{16}(CH_3OH)_2(OH)_2$}·$3CHCl_3$]$_\infty$ (**10**·$3CHCl_3$).

**Bond distances (Å)**			
Ru1–N11	2.058(4)	Zn1–N21	2.063(4)
Ru1–N12	2.060(4)	Zn3–N22	2.363(4)
Ru1–P21	2.322(2)		
**Bond angles (°)**			
N11–Ru1–N12	90.2(2)	O33–Zn1–N21	133.4(2)
N11–Ru1–P21	92.6(1)	O310–Zn3–N22	80.6(2)
N12–Ru1–P21	89.9(1)	O35–Zn3–N22	81.6(1)
O317–Zn1–N21	98.3(2)	O314–Zn3–N22	82.19(2)
O32–Zn1–N21	99.6(2)	O316–Zn3–N22	175.1(1)
**Dihedral angles (°)**			
Ru(TPP)··· Ph1	63.3(2)	Ru(TPP)··· Ph2	65.6(2)

Table S8bis. Selected coordination distances (Å) and angles (°) for the zinc-acetate cluster part of [{Ru(TPP)(PTA-κ^3*P*,*2N*)$_2$}{$Zn_9(CH_3COO)_{16}(CH_3OH)_2(OH)_2$}·$3CHCl_3$]$_\infty$ (**10**·$3CHCl_3$).

**Bond distances (Å)**			
Zn1–O317	1.962(4)	Zn3–O316	2.039(3)
Zn1–O32	1.936(6)	Zn3–O35	2.009(4)
Zn1–O33	1.960(4)	Zn4–O311	2.170(4)
Zn2–O31	2.073(4)	Zn4–O313	2.126(4)
Zn2–O315	2.077(4)	Zn4–O316	2.015(3)
Zn2–O318	2.074(4)	Zn5–O312	1.983(4)
Zn2–O33	2.121(5)	Zn5–O313	2.473(4)
Zn2–O36	2.087(4)	Zn5–O316	1.958(4)
Zn2–O38	2.081(4)	Zn5–O37	1.990(4)
Zn3–O310	1.984(4)	Zn5–O38	2.570(4)
Zn3–O314	1.971(3)	Zn5–O39	2.040(4)
**Bond angles (°)**			
O32–Zn1–O33	100.9(2)	O314–Zn3–O35	116.8(2)
O32–Zn1–O317	113.0(2)	O310–Zn3–O35	117.5(2)
O33–Zn1–O317	111.1(2)	O314–Zn3–O310	119.1(2)
O38–Zn2–O36	83.0(2)	O313–Zn4–O311	90.7(2)
O315–Zn2–O36	84.8(2)	O316–Zn4–O311	90.2(1)
O318–Zn2–O315	85.4(2)	O316–Zn4–O313	97.5(1)
O315–Zn2–O38	89.4(2)	O312–Zn5–O313	84.5(2)
O31–Zn2–O33	89.4(2)	O312–Zn5–O37	104.7(2)

O38–Zn2–O33	90.0(2)	O312–Zn5–O39	97.4(2)
O31–Zn2–O318	90.3(2)	O316–Zn5–O312	111.3(2)
O31–Zn2–O315	91.1(2)	O316–Zn5–O313	75.1(1)
O318–Zn2–O38	93.1(2)	O316–Zn5–O37	140.1(2)
O31–Zn2–O36	93.7(2)	O316–Zn5–O39	101.1(1)
O36–Zn2–O33	94.1(2)	O37–Zn5–O313	92.2(1)
O318–Zn2–O33	95.7(2)	O37–Zn5–O39	90.5(2)
O318–Zn2–O36	169.5(1)	O38–Zn5–O312	156.2(2)
O31–Zn2–O38	176.7(2)	O38–Zn5–O313	83.5(1)
O315–Zn2–O33	178.8(2)	O38–Zn5–O316	85.3(1)
O35–Zn3–O316	94.5(1)	O38–Zn5–O37	55.4(2)
O310–Zn3–O316	98.8(2)	O38–Zn5–O39	95.9(2)
O314–Zn3–O316	102.3(2)	O39–Zn5–O313	176.2(1)

Table S9. Selected coordination distances (Å) and angles (°) for [{Ru(TPP)(PTA-κ*P*)(PTA-κ^2*P*,*N*)}{$ZnCl_2(OH_2)$}·0.6$CHCl_3$] (**11**·0.6$CHCl_3$).

Bond distances (Å)			
Ru1–N21	2.049(3)	Zn1–O1[a]	2.10(1)
Ru1–N22	2.052(3)	Zn1–Cl1[a]	2.23(1)
Ru1–P1	2.310(1)	Zn1–Cl3[a]	2.367(2)
Zn1–N11[a]	2.192(7)		
**Bond angles (°)**			
N21–Ru1–P1	88.96(9)	O1–Zn1–Cl1[a]	120.6(7)
N22–Ru1–P1	90.2(1)	O1–Zn1–Cl3[a]	101.2(7)
N21–Ru1–N22	90.3(1)	O1–Zn1–N11[a]	113.7(4)
Cl1–Zn1–Cl3[a]	115.0(7)	Cl3–Zn1–N11[a]	99.7(5)
Cl1–Zn1–N11[a]	104.5(5)		
**Dihedral angles (°)**			
Ru(TPP)··· Ph1	73.5(2)	Ru(TPP)··· Ph2	72.1(1)

[a] values averaged over the two populations of the {$ZnCl_2(OH_2)$} group
